# Interplay of the Serine/Threonine-Kinase StkP and the Paralogs DivIVA and GpsB in Pneumococcal Cell Elongation and Division

**DOI:** 10.1371/journal.pgen.1004275

**Published:** 2014-04-10

**Authors:** Aurore Fleurie, Sylvie Manuse, Chao Zhao, Nathalie Campo, Caroline Cluzel, Jean-Pierre Lavergne, Céline Freton, Christophe Combet, Sébastien Guiral, Boumediene Soufi, Boris Macek, Erkin Kuru, Michael S. VanNieuwenhze, Yves V. Brun, Anne-Marie Di Guilmi, Jean-Pierre Claverys, Anne Galinier, Christophe Grangeasse

**Affiliations:** 1 Bases Moléculaires et Structurales des Systèmes Infectieux, IBCP, Université Lyon 1, CNRS, UMR 5086, Lyon, France; 2 Key laboratory of Medical Molecular Virology, School of Basic Medical Sciences, Shanghai Medical College, Fudan University, Shanghai, China; 3 Centre National de la Recherche Scientifique, LMGM-UMR5100, Toulouse, France; 4 Université de Toulouse, UPS, Laboratoire de Microbiologie et Génétique Moléculaires, Toulouse, France; 5 Laboratoire de Biologie Tissulaire et d'Ingénierie Thérapeutique, IBCP, Université Lyon 1, CNRS, UMR5305, Lyon, France; 6 Proteome Center Tuebingen, Interdepartmental Institute for Cell Biology, University of Tuebingen, Tuebingen, Germany; 7 Departments of Biology and Chemistry, Indiana University, Bloomington, Indiana, United States of America; 8 Institut de Biologie Structurale, UMR 5075, Université Joseph Fourier, CNRS, CEA, Grenoble, France; 9 Laboratoire de Chimie Bactérienne, UMR7283, IMM, CNRS, Aix-Marseille Université, Marseille, France; University of Geneva Medical School, Switzerland

## Abstract

Despite years of intensive research, much remains to be discovered to understand the regulatory networks coordinating bacterial cell growth and division. The mechanisms by which *Streptococcus pneumoniae* achieves its characteristic ellipsoid-cell shape remain largely unknown. In this study, we analyzed the interplay of the cell division paralogs DivIVA and GpsB with the ser/thr kinase StkP. We observed that the deletion of *divIVA* hindered cell elongation and resulted in cell shortening and rounding. By contrast, the absence of GpsB resulted in hampered cell division and triggered cell elongation. Remarkably, Δ*gps*B elongated cells exhibited a helical FtsZ pattern instead of a Z-ring, accompanied by helical patterns for DivIVA and peptidoglycan synthesis. Strikingly, *divIVA* deletion suppressed the elongated phenotype of Δ*gps*B cells. These data suggest that DivIVA promotes cell elongation and that GpsB counteracts it. Analysis of protein-protein interactions revealed that GpsB and DivIVA do not interact with FtsZ but with the cell division protein EzrA, which itself interacts with FtsZ. In addition, GpsB interacts directly with DivIVA. These results are consistent with DivIVA and GpsB acting as a molecular switch to orchestrate peripheral and septal PG synthesis and connecting them with the Z-ring *via* EzrA. The cellular co-localization of the transpeptidases PBP2x and PBP2b as well as the lipid-flippases FtsW and RodA in Δ*gps*B cells further suggest the existence of a single large PG assembly complex. Finally, we show that GpsB is required for septal localization and kinase activity of StkP, and therefore for StkP-dependent phosphorylation of DivIVA. Altogether, we propose that the StkP/DivIVA/GpsB triad finely tunes the two modes of peptidoglycan (peripheral and septal) synthesis responsible for the pneumococcal ellipsoid cell shape.

## Author Summary

Over the last decade, bacterial genomics have revealed the presence of eukaryotic-type serine/threonine protein kinases (STKPs) in many bacteria. However, their role and mode of action is still elusive. Recent studies have suggested that STKPs could play an important role in regulating cell division of some bacterial species but the underlying regulatory mechanisms are largely unknown. Considering that much remains to be discovered about the mechanisms by which the cell division machinery is assembled at the cell center and how the diversity of bacterial cell shapes is achieved and maintained, studying the role of STKPs represents a promising approach to decipher the inner workings of bacterial cell division. In this article, we show that the ser/thr-kinase StkP and the two cell division paralogs GpsB and DivIVA of *Streptococcus pneumoniae* (the pneumococcus) work together to finely tune peptidoglycan synthesis and achieve proper cell shape and division. We discuss the likelihood that similar mechanisms occur in other bacteria requiring protein-kinases for the cell division process. We propose that the interplay between protein-kinases and cell-division proteins like GpsB or DivIVA is of crucial importance to satisfy the modes of cell division and the cell shape displayed by streptococci and other bacteria.

## Introduction

Bacterial cell growth and division are intimately linked. Complex webs of proteins interacting with each other temporally and spatially control the cellular events leading to the production of two identical daughter cells [1]–[3]. Most of the proteins required for cell division and elongation have been characterized in rod-shaped bacterial models like the Gram-negative bacteria *Escherichia coli* and *Caulobacter crescentus* or the Gram-positive bacterium *Bacillus subtilis*, and robust models depicting their division process are proposed. This knowledge has been beneficial for characterizing and understanding cell division of other bacteria. However, some aspects related to cell division, including the achievement of cell shape, are often hardly transposable and species-specific mechanisms exist to allow cells to divide, assume a given shape and/or cope with their environment [4], [5].

In the Gram-positive human pathogen *Streptococcus pneumoniae* (the pneumococcus), some conserved division proteins have been studied, but overall, little is known about the mechanisms governing cell division and those responsible for peptidoglycan (PG) synthesis, as well as how this species achieves its characteristic ellipsoid (rugby-ball like) shape [6]–[10]. Early studies have suggested that *S. pneumoniae* utilizes a combination of two PG synthesis modes, namely septal and peripheral [11]. Due to the absence of the actin-like protein MreB and any homologues in the pneumococcus, it is speculated that these two modes of PG synthesis are coordinated with and organized by FtsZ-ring formation [12]. A two-state model in which two different PG synthesis machineries are responsible for either septal or peripheral synthesis has been proposed. In this model, the PG transpeptidase PBP2x, a penicillin binding protein (PBP) that catalyzes PG cross-linking, and the lipid-flippase FtsW, that transports lipid-linked PG precursors from the inner to the outer leaflet of the cytoplasmic membrane, belong to a septal machinery and are exclusively required for septal PG synthesis and cell separation. On the other hand, the transpeptidase PBP2b and the lipid-flippase RodA would be exclusively associated with a peripheral machinery, and required for peripheral PG synthesis and cell elongation. However, it is unclear how *S. pneumoniae* would coordinate peripheral and septal synthesis. An interesting possibility comes from work in *B. subtilis* showing that cell elongation-division cycles are controlled by shuttling of PBP1, a transpeptidase/transglycosidase class A PBP involved in peptidoglycan polymerization [13]. PG synthesis could thus be fine-tuned by a yet uncharacterized process to allow the alternate synthesis of septal and peripheral PG in pneumococcus. StkP, a membrane eukaryotic-type serine/threonine kinase, represents an attractive candidate to regulate septal and peripheral PG synthesis in *S. pneumoniae*. This kinase has been recently shown to play an important role in pneumococcal cell division and growth [14]–[16]. *In vivo*, only a few proteins appear specifically phosphorylated by StkP [14], [17], [18]. Among them, it is noteworthy to find the division protein DivIVA, which was shown to be phosphorylated by StkP on Thr-201. Expression of the non-phosphorylatable form of DivIVA (i.e., a mutant in which Thr-201 is substituted for an alanine) induced severe defects in cell shape, possibly by affecting pole maturation [14].

Interestingly, a DivIVA paralog, named GpsB [19], was identified in *B. subtilis* and shown to be involved together with EzrA in PBP1 shuttling between elongation and division sites [13]. Global phosphoproteome analyses of *B. subtilis* and *Streptococcus agalactiae* indicated that GpsB is phosphorylated in these species [20], [21]. GpsB is also found in *S. pneumoniae*, as in most Firmicutes. This situation prompted us to investigate the role of the two paralogs, GpsB and DivIVA, in PG synthesis and cell morphogenesis of *S. pneumoniae*, and to examine whether their phosphorylation by StkP could affect their role. Here, we establish that GpsB and DivIVA are both crucial for cell morphogenesis, and demonstrate that DivIVA is necessary for cell elongation whereas GpsB acts as a negative regulator of DivIVA to prevent cell elongation. Moreover, we show that GpsB is not phosphorylated, but required for StkP septal localization and subsequent phosphorylation of DivIVA. In light of these observations, we propose that the StkP/DivIVA/GpsB triad finely tunes the two modes of PG synthesis to achieve the ovoid shape of pneumococci and we discuss the relevance of this process in other bacteria. Our observation of similar localization patterns for the transpeptidases PBP2x and PBP2b as well as for the lipid-flippases FtsW and RodA in cells deficient for GpsB and/or DivIVA questions the existence of two distinct PG biosynthesis machineries.

## Results

### Inactivation of *divIVA* hampers cell elongation

**Figure 1:**
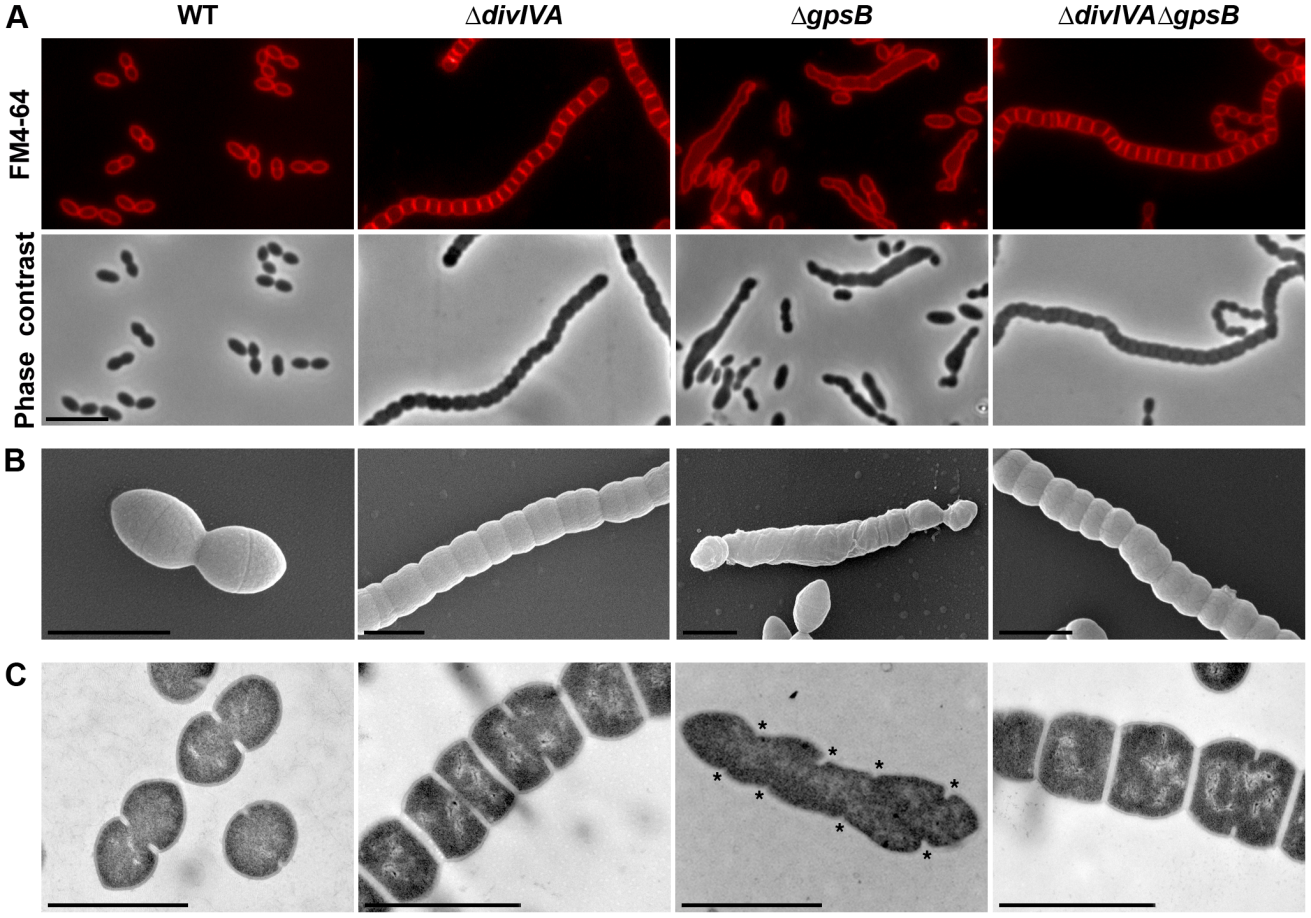
Morphology of WT, Δ*divIVA*, Δ*gpsB* and Δ*divIVA*Δ*gpsB* cells. (A) Phase contrast microscopy (lower panel) and FM4–64 membrane staining (upper panel) images of WT, Δ*divIVA*, Δ*gpsB* and Δ*divIVA*Δ*gpsB* exponentially growing cells at 37°C in THY medium. Scale bar, 5 µm. (B) Scanning electron micrograph of WT, Δ*divIVA*, Δ*gpsB* and Δ*divIVA*Δ*gpsB* cells. Scale bar, 1 µm. (C) Transmission electron micrograph of WT, Δ*divIVA*, Δ*gpsB* and Δ*divIVA*Δ*gpsB* cells. Scale bar, 1 µm. Asterisks indicate defective septal initiations in staggered rows in the Δ*gpsB* cell.

To analyze the potential role of DivIVA in pneumococcal morphogenesis, we constructed a nonpolar, markerless *divIVA*-null mutant and investigated its cell morphology. As previously reported for the *S. pneumoniae* RX1 strain [6], [22], 99.8% of Δ*divIVA* R800 cells exhibited a striking chain phenotype (Figure 1A and Table S1). When the *divIVA* mutation was repaired back to wild type (WT) by transformation, the morphology of the resulting strain was similar to that of the WT strain with a typical diplo-ovococcal shape (compare Figure 1A with Figure S1) indicating that the chain phenotype is due to the deletion of *divIVA*. Δ*divIVA* chains contained up to several dozen of tightly joined cells separated by well-defined membranes (Figures 1A and S2A). Cells were clearly not ovoid but flattened at the poles, exhibiting a rounded shape. Analysis of individual cell parameters further confirmed this visual impression and showed that *divIVA* deletion resulted in reduced pneumococcal cell length (Figure S2B and Table S1).

**Figure 2:**
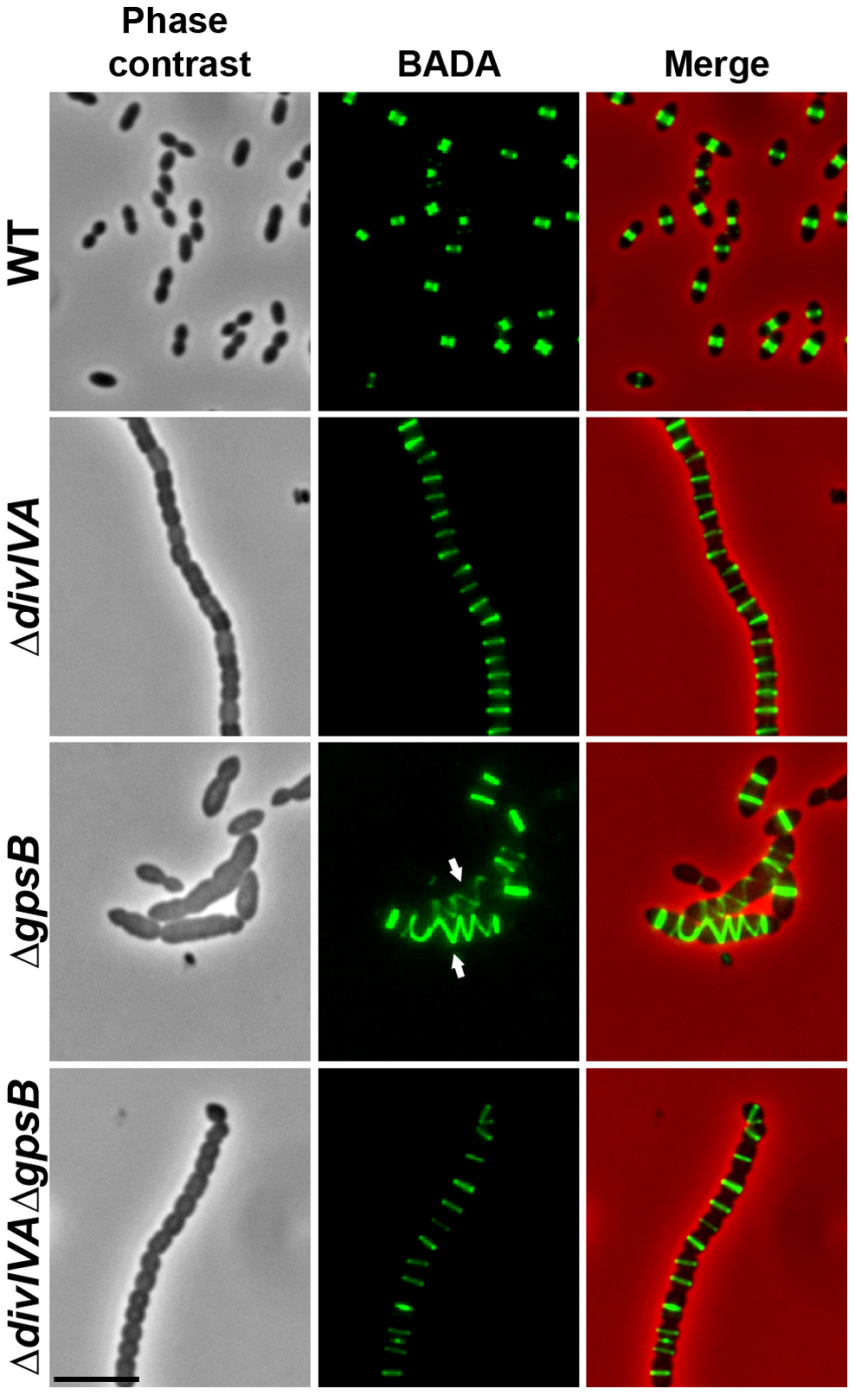
Localization of PG synthesis in WT, Δ*divIVA*, Δ*gpsB* and Δ*divIVA*Δ*gpsB* cells. Phase contrast microscopy (left panel) and BADA labeling of PG (middle panel) images of WT, Δ*divIVA*, Δ*gpsB* and Δ*divIVA*Δ*gpsB* exponentially growing cells pulsed with BADA 4 min each at 37°C in THY medium. Overlay between phase contrast (red) and BADA (green) labeling is shown. Arrows show helical organization of PG synthesis. Scale bar, 5 µm.

We also examined Δ*divIVA* cells by scanning- and transmission-electron microscopy (SEM and TEM). Using SEM, cells seemed to be interlocked into the neighboring ones (Figure 1B). Nevertheless, TEM indicated that cells were clearly separated by membranes, consistent with efficient Z-ring constriction and closure, and suggesting that septal PG is efficiently produced (Figure 1C). To confirm the latter, we applied the strategy described by Kuru and co-workers [23] and PG synthesis was visualized using Bodipy-FL containing fluorescent D-amino acid, namely Bodipy-FL-amino-D-alanine, or BADA [24]. More specifically, the exponentially growing cells are pulsed with BADA for 4 min corresponding to ca. 10–12% of the generation times of the WT and the mutants. As a control, we checked that BADA labeled the division site in WT cells as previously described using fluorescent vancomycin [14] (Figure 2). BADA labeling of Δ*divIVA* cells revealed PG synthesis localizing exclusively as bands across the cells at the division septa in 99.4% of cells (Figure 2 and Table S2). Altogether, these results suggest that in the absence of DivIVA, cell elongation is hampered while septum closure still occurs. On the other hand, the last step of cell division allowing the final separation of daughter cells is affected.

### Inactivation of *gpsB* hinders cell septation

To unravel the role of GpsB in *S. pneumoniae*, we first constructed a nonpolar markerless deletion mutant of *gpsB* in strain R800. In contrast to the deletion of *divIVA*, the deletion of *gpsB* severely affected growth (Figure S3A) and cell viability decreased to 60% suggesting that GpsB is crucial for the pneumococcus. Microscopy analysis revealed a striking phenotype characterized by the presence of very elongated cells (Figures 1A, S3B and Table S1). Morphometric measurements indicated that the length was below 1.4 µm for 90% of wild-type cells (WT), whereas nearly 90% of Δ*gpsB* cells exhibited length greater than 1.3 µm. Δ*gpsB* cells seemed irregularly shaped and septal membranes across cells were lacking, indicating that cell constriction was seriously hampered (Figure 1A). Examination of the ultrastructure of Δ*gpsB* cells by TEM and SEM confirmed that mutant cells displayed a strongly affected morphology with irregular width (Figures 1B–C). The presence of several septal initiations positioned asymmetrically on each side of the long axis of the cells was detected by TEM (Figure 1C). SEM images confirmed an irregular elongation of Δ*gpsB* cells which displayed a “twisted-towel” shape (Figure 1B). One could further observe the presence of a helical groove at the surface of the cells that seemed to correspond to the asymmetric septal initiations detected by TEM, suggesting that the divisome is stretched upon cell elongation. Importantly, a wild-type diplo-ovococcal shape and normal growth were restored when the Δ*gpsB* strain was transformed back to *gpsB^+^* confirming that the observed phenotype resulted from the inactivation of *gpsB* (compare Figure 1A with Figure S1). Deletion of *gpsB* was also attempted into four other well-characterized and widely used *S. pneumoniae* strains, the encapsulated D39 and TIGR4, and the unencapsulated R6 and RX1. The same elongated phenotype was observed with *gpsB^−^* derivatives of the unencapsulated strains, while our efforts to delete *gpsB* failed with both encapsulated strains (Figure S3C). The latter observation is consistent with the recent report of Land and co-workers [25]. Altogether, these data show that deletion of *gpsB* triggers cell elongation and prevents proper pneumococcal cell division.

### Helical FtsZ pattern in Δ*gpsB* cells

**Figure 3:**
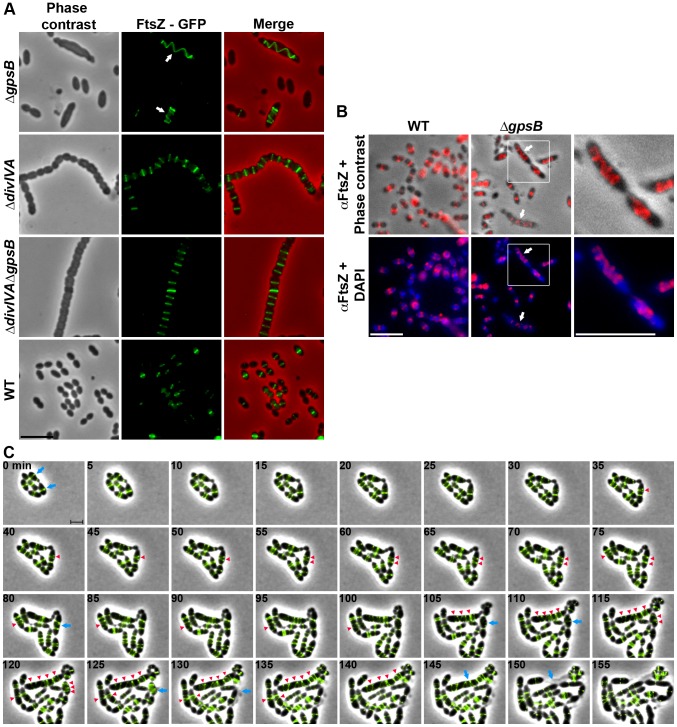
FtsZ localization in WT, Δ*divIVA*, Δ*gpsB* and Δ*divIVA*Δ*gpsB* cells. (A) FtsZ localization in WT, Δ*divIVA*, Δ*gpsB* and Δ*divIVA*Δ*gpsB* cells. Phase contrast (left), GFP fluorescent signal (middle) and overlays (right) between phase contrast (red) and GFP (green) images are shown. Arrows show helical organization of FtsZ. Scale bar, 5 µm. See also the unprocessed image of FtsZ localization in Δ*gpsB* cells in Figure S5 showing that the FtsZ fluorescent signal is detected in all cells. (B) Immunofluorescence staining of fixed WT and Δ*gpsB* cells using anti-FtsZ polyclonal antibodies. DNA was counterstained with DAPI. Merged pictures show (upper panels) the overlay of FtsZ (red) and phase contrast images, and (lower panels) the overlay of FtsZ (red) and DAPI (blue). Higher magnifications of Δ*gpsB* cells highlighted with a white square are shown in the right row. Arrows show helical organization of FtsZ. Scale bar, 5 µm. (C) Fluorescence time-lapse microscopy of Δ*gpsB* cells producing FtsZ-GFP and grown in C+H medium at 30°C. Overlays between phase contrast (gray) and GFP (green) are shown. Stills are from Movie S2. Scale bar, 2 µm. Blue arrows point to cells in which the Z-ring helix-stretches until cell death. Red arrowheads point to helical structures of FtsZ. FtsZ-GFP is the only source of FtsZ in cells. *ftsZ-gfp* substitutes the native *ftsZ* gene at its chromosomal locus.

We then analyzed the effect of the deletion of *gpsB* on FtsZ localization. For this purpose, we first constructed a C-terminal GFP fusion to FtsZ. Throughout this study and unless otherwise indicated, C-terminal and N-terminal fusions (denoted respectively Protein-GFP and GFP-Protein) were constructed at each native chromosomal locus, expressed under the control of the native promoter and represented the only source of protein. The FtsZ-GFP fusion seemed fully functional as cells grew as rapidly as WT cells and did not display any shape defect (Figures 3A and S4A). As expected, FtsZ-GFP localized at midcell in exponentially grown WT cells as well as in Δ*divIVA* rounded cells (Figure 3A and Table S2). FtsZ-GFP also appeared as several transversal bands in a majority of Δ*gpsB* cells. Interestingly however, a number of cells (25.2%) displayed a zig-zag localization of FtsZ (Figures 3A, S5 and Table S2). To clarify the dimensional nature of this zig-zag structure, we carried out deconvolution microscopy (Movie S1) that revealed a continuous FtsZ-helical organization. This unexpected helical localization of FtsZ in Δ*gpsB* cells was confirmed by immunostaining using anti-FtsZ antibodies (Figure 3B) indicating that the helical pattern is not an artifact due to the GFP tag on FtsZ. Control experiments also confirmed that FtsZ levels were unaffected in the absence of GpsB (Figure S4B). To reconcile the dual FtsZ localization (spirals *vs.* bands) in Δ*gpsB* elongated cells, time-lapse microscopy was performed. During cell elongation, some Z-rings were replaced by helical structures which eventually split to generate several new Z-rings, whereas others continued to stretch (Figure 3C and Movie S2). However, in both cases cells ended up bursting and dying (e.g., cells marked with arrows in Figure 3C). We conclude from these observations that the Z-ring is replaced by a helical structure during elongation of Δ*gpsB* cells.

### Helical PG synthesis, PBP2x, PBP2b, FtsW and RodA pattern in Δ*gpsB* cells

**Figure 4:**
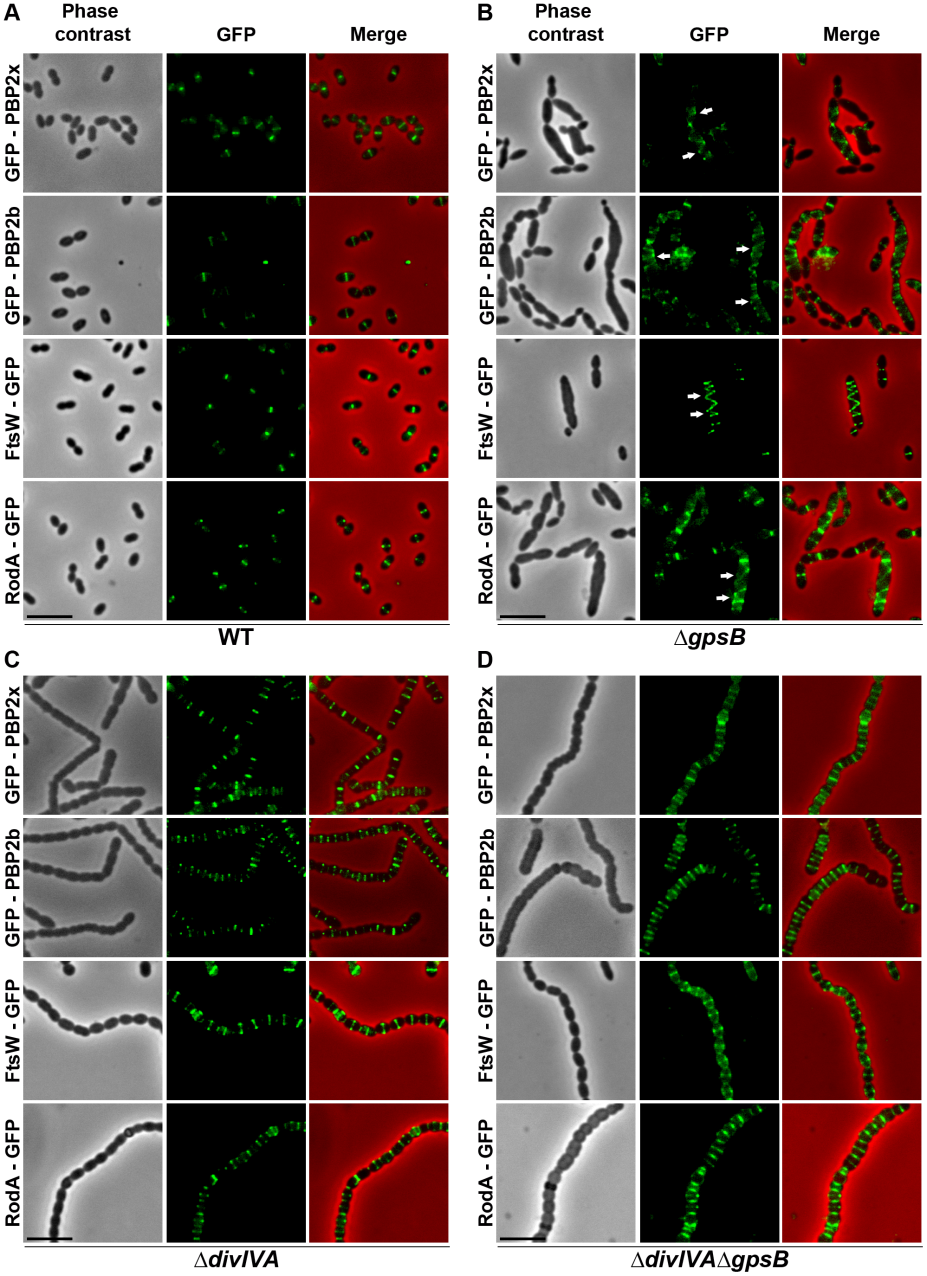
Localization of PBP2x, PBP2b, FtsW and RodA in WT, Δ*divIVA*, Δ*gpsB* and Δ*divIVA*Δ*gpsB* cells. Chromosomal copy of either *pbp2x*, *pbp2b*, *ftsW* or *rodA* were substituted for a *gfp*-fused gene in WT (A) or Δ*gpsB* (B), or Δ*divIVA* (C), or Δ*divIVA*Δ*gpsB* (D) cells. Cells were grown in THY medium at 37°C. GFP (green) and phase-contrast (grey) images were taken from a typical field of exponentially growing cells. Merged pictures show the overlay of GFP fluorescence (green) and phase contrast images (red). Arrows show helical organization of GFP-PBP2x, GFP-PBP2b, FtsW-GFP and RodA-GFP. Scale bar, 5 µm. All fusion proteins are the only source of PBP2X, PBP2b, FtsW or RodA in the cells.

To test whether cell elongation in Δ*gpsB* cells was accompanied by altered localization of PG synthesis, the latter was labeled using BADA. BADA labeling revealed a helical organization of neosynthesized PG in 28.7% of Δ*gpsB* elongated cells (Figure 2 and Table S2), which is comparable to the percentage of cells exhibiting GFP-FtsZ spirals. This prompted us to examine the localization of PBP2x, PBP2b, FtsW and RodA, which are involved in PG synthesis, using GFP-PBP2x, GFP-PBP2b, FtsW-GFP or RodA-GFP. All four GFP-fused proteins were functional as cells grew normally and displayed WT shape (Figures 4A and S6A). Fluorescence microscopy indicated that PBP2x, PBP2b, FtsW, and RodA localize at midcell in WT cells as well as in Δ*divIVA cells* (Figures 4A and 4C). Strikingly, they all appeared mislocalized in elongated Δ*gpsB* cells, exhibiting a helical pattern (Figure 4B and Table S2) reminiscent of that observed for FtsZ (Figure 3A) and PG synthesis (Figure 2). Western blot control experiments confirmed that the four GFP-fusions were produced at similar levels in WT, Δ*divIVA* and Δ*gpsB* cells, excluding any artifact due to aberrant protein expression (Figure S7).

**Figure 5:**
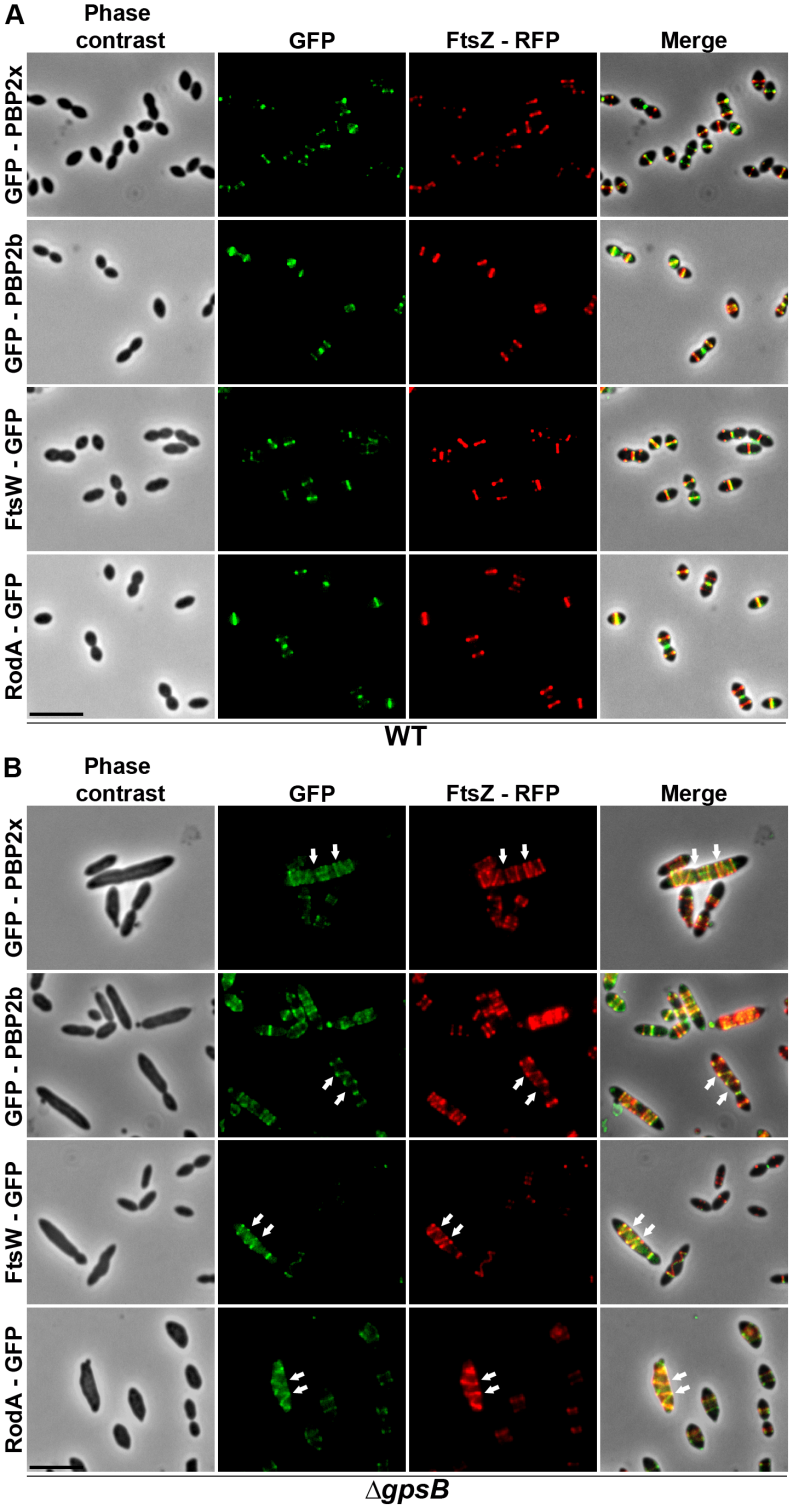
Localization of GFP fused PBP2x, PBP2b, FtsW or RodA together with FtsZ-RFP in WT and Δ*gpsB* cells. Localization of FtsZ-RFP and either GFP-PBP2x, GFP-PBP2b, FtsW-GFP or RodA-GFP in WT (A) or Δ*gpsB* (B) cells grown at 37° in THY. Overlays between phase contrast (gray), GFP (green), and RFP (red) are shown on the right. Arrows show helical organization of FtsZ-RFP, GFP-PBP2x, GFP-PBP2b, FtsW-GFP and RodA-GFP. Scale bar, 5 µm. All fusion proteins are the only source of FtsZ, PBP2X, PBP2b, FtsW or RodA in cells. The fusion genes encoding these proteins substitute the corresponding native genes at their chromosomal locus.

These helical patterns suggested that the four GFP-proteins could co-localize with FtsZ. To directly assess this, we constructed double-labeled strains containing FtsZ fused to RFP and either PBP2x, PBP2b, FtsW, or RodA fused to GFP. Cells containing a pair of fusion proteins in an otherwise WT background exhibited a growth delay (Figure S6B) indicating that the combination of FtsZ-RFP with GFP-fused PBP2x, PBP2b, FtsW, or RodA is somehow detrimental. Nevertheless, microscopy analyses indicated that cell shapes were normal and that each pair of RFP/GFP-fusions co-localized properly at midcell (Figure 5A). On the other hand, when we detected helical RFP-FtsZ in Δ*gpsB* elongated cells, PBP2x, PBP2b, FtsW and RodA also displayed an helical organization co-localizing with helical FtsZ (Figure 5B).

### Helical DivIVA pattern in Δ*gpsB* cells

**Figure 6:**
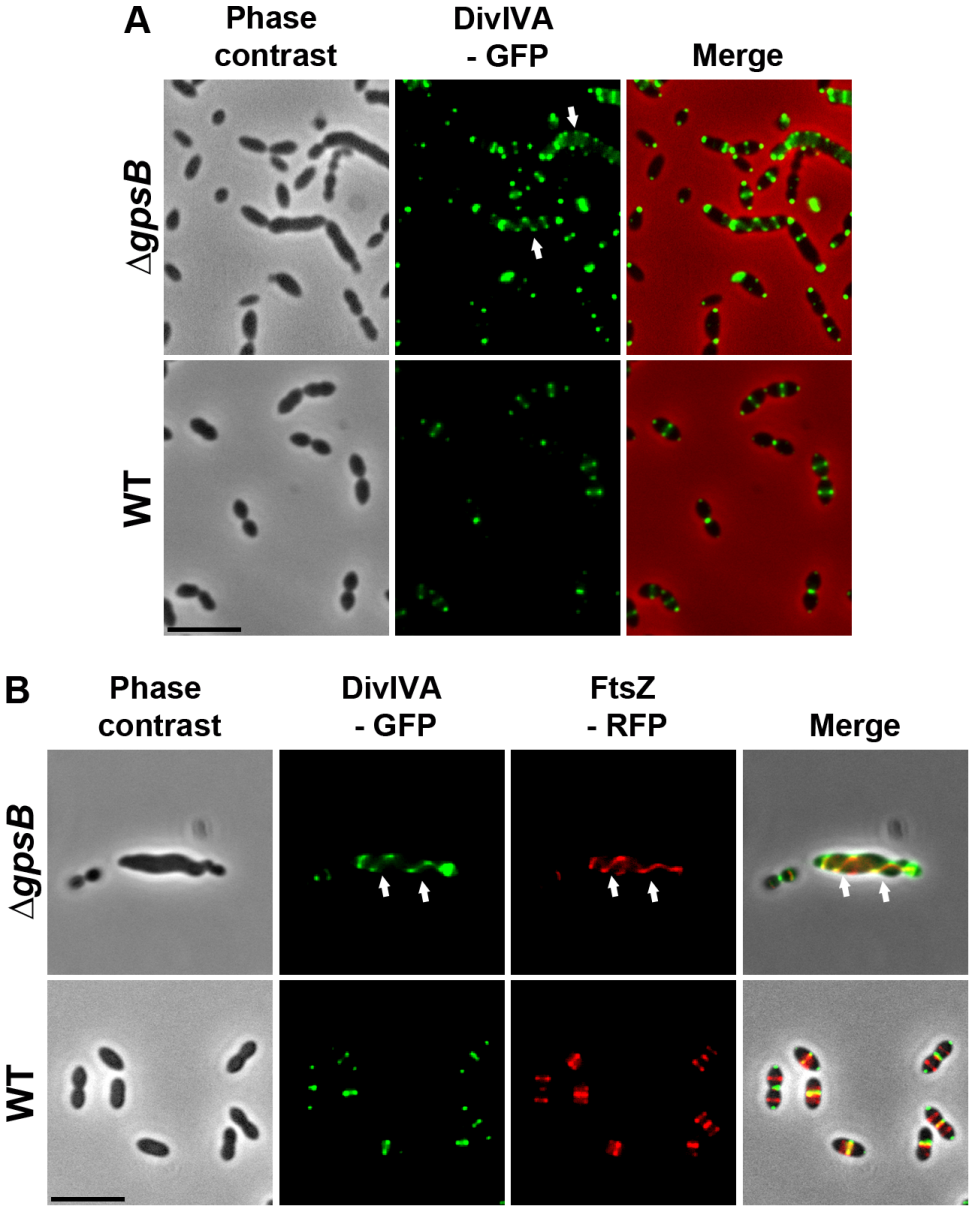
Localization of DivIVA in WT and Δ*gpsB* cells. (A) DivIVA-GFP localization in WT and Δ*gpsB* cells. Phase contrast (left), GFP fluorescent signal (middle) and overlays (right) between phase contrast (red) and GFP (green) images are shown. (B) Co-localization of FtsZ-RFP (red) and DivIVA-GFP (green) in WT and Δ*gpsB* cells. Overlays between phase contrast (gray), GFP (green), and RFP (red) are shown. Cells were grown to exponential phase in THY medium at 37°C. Arrows show helical organization of DivIVA-GFP and FtsZ-RFP. Scale bar, 5 µm. DivIVA-GFP and FtsZ-GFP are the only source of FtsZ and DivIVA in cells. *ftsZ-gfp* and *divIVA-gfp* substitute the native *ftsZ* and *divIVA* genes at their chromosomal locus, respectively.

To examine DivIVA localization in Δ*gpsB* elongated cells, we first generated a WT strain producing DivIVA-GFP. WT cells expressing DivIVA-GFP grew normally and displayed a classic ovoid-shape (Figures 6A and S8A) establishing that the DivIVA-GFP fusion is functional. In agreement with a previous report [15], DivIVA-GFP localized at both midcell and the cell poles (Figure 6A). By contrast, DivIVA-GFP exhibited a helical organization in 20.1% of elongated Δ*gpsB* cells (Figure 6A and Table S2). This phenotype was not due to an aberrant expression of DivIVA-GFP since western blot analyses confirmed that the fusion protein was synthesized at similar levels in WT and Δ*gpsB* cells (Figure S8B). DivIVA localization was thus comparable to that of FtsZ in Δ*gpsB* cells (Figure 3A) suggesting that the two proteins co-localize during cell elongation. To confirm this, we constructed WT and Δ*gpsB* strains expressing both DivIVA-GFP and FtsZ-RFP. As expected, FtsZ and DivIVA displayed respectively septal and septal/polar localization in WT cells (Figure 6B). In Δ*gpsB* cells, DivIVA co-localized with FtsZ in a helical pattern (Figure 6B).

### Inactivation of *divIVA* suppresses elongation and helical patterns of Δ*gpsB* cells

To further investigate the role of GpsB and DivIVA, we introduced the *divIVA* deletion in cells deficient for *gpsB*. The double mutant was readily obtained. FM4–64 membrane staining showed that Δ*divIVA*Δ*gpsB* cells exhibited the same cell shape and chain phenotype as Δ*divIVA* cells (97.9% of cells), although a few cells were irregularly shaped (Figure 1A and Table S1), indicating that inactivation of *divIVA* suppressed the elongated cell phenotype typical of Δ*gpsB* cells (the same was observed with a double mutant constructed by introducing the Δ*gpsB* mutation into Δ*divIVA* cells; data not shown). However, cell viability decreased to 60% and was comparable to that of Δ*gpsB* cells. As controls, FtsZ-GFP still localized at the division septa in the absence of DivIVA and GpsB, and was produced to WT levels (Figure 3A, Table S2 and compare Figures S4B with S9B). These observations suggest that GpsB interplays with DivIVA to coordinate cell elongation and cell division, and that GpsB is dispensable for septal PG synthesis when DivIVA is absent.

To further test this hypothesis, we analyzed BADA labeling of PG in Δ*divIVA*Δ*gpsB* cells. We observed that PG is produced properly at the division site (Figure 2 and Table S2). Likewise, we analyzed the localization of PBP2x, PBP2b, FtsW and RodA in Δ*divIVA*Δ*gpsB* cells (Figure 4D and Table S2). All of them still localized to the division septa as observed in Δ*divIVA* cells (Figure 4C and Table S2). Western blot control experiments confirmed that the four GFP-fused proteins were produced at similar levels in WT and Δ*divIVA*Δ*gpsB* cells (Figure S7). These observations indicate that deletion of *gpsB* is tolerated in a Δ*divIVA* mutant without inducing further detectable cell shape and septum closure defects and does not impair PG synthesis at the division site.

### Interconnections between GpsB, DivIVA and the Z-ring

To gain an insight into a possible connection between GpsB, DivIVA and cell division, we first looked for physical interactions between these proteins and FtsZ using a bacterial two-hybrid screen [26]. Neither GpsB nor DivIVA were found to interact with FtsZ (Figure S10A). However, as the cell division protein EzrA was found to bridge GpsB with the Z-ring in *B. subtilis* [13], we also analyzed EzrA interactions with GpsB, DivIVA and FtsZ. Reproducible interactions were detected between EzrA and either GpsB, DivIVA or FtsZ by bacterial two-hybrid assays (Figure S10A). These interactions were further analyzed by surface plasmon resonance (SPR), which confirmed that EzrA interacts with GpsB (K_D_ = 770±230 nM), DivIVA (K_D_ = 530±75 nM) and FtsZ (K_D_ = 295±60 nM) (Figures S10B and S10C–E). We also tested whether GpsB interacts with DivIVA. Reproducible interactions were first detected with the two-hybrid screen (Figure S10A) and SPR confirmed that GpsB interacts with DivIVA (K_D_ = 85±14 nM) (Figures S10B and S10F).

**Figure 7:**
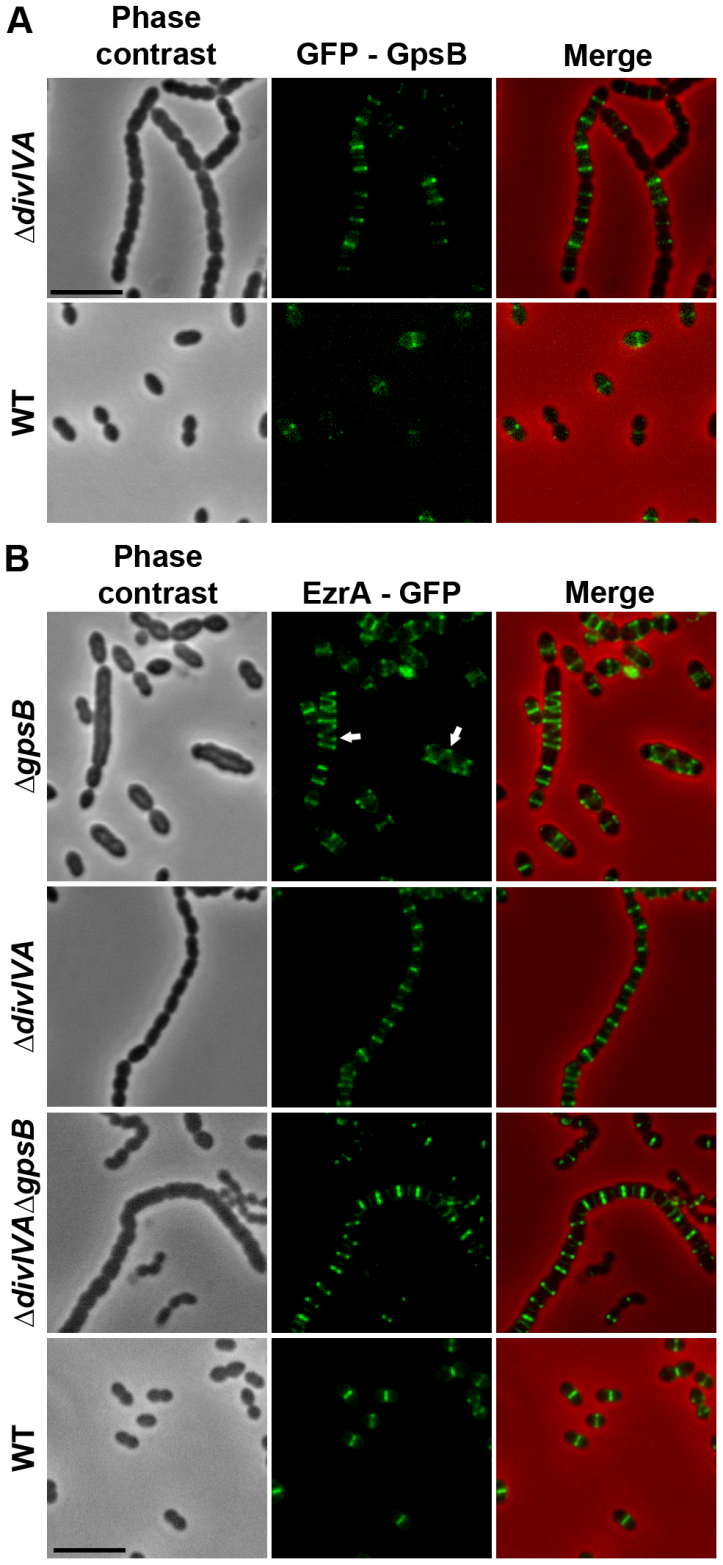
Localization of GpsB and EzrA. (A) Localization of GFP-GpsB in WT and Δ*divIVA* cells. Expression of the *gfp*-*gpsB* fusion is under the control of the zinc-inducible P*_zn_* promoter at the non-essential *bgaA* locus. (B) EzrA-GFP localization in WT, Δ*gpsB*, Δ*divIVA* and Δ*divIVA*Δ*gpsB* cells. Phase contrast (left), GFP fluorescent signal (middle) and overlays (right) between phase contrast (red) and GFP (green) images are shown. Cells were grown to exponential phase in THY medium at 37°C. Arrows show helical organization of EzrA-GFP. Scale bar, 5 µm. EzrA-GFP is the only source of EzrA in cells. *ezrA-gfp* substitutes the native *ezrA* gene at its chromosomal locus.

We then analyzed the localization of EzrA and GpsB fused to GFP. WT cells producing GFP-GpsB or GpsB-GFP appeared elongated and displayed aberrant cell shapes indicating that both fusions were not fully functional (Figure S11). We therefore constructed a merodiploid strain carrying an ectopic *gfp*-*gpsB* fusion under the control of the zinc-inducible P_Zn_ promoter at the non-essential *bgaA* locus. Fluorescence microscopy indicated that GFP-GpsB localizes as bands across the short axis of the cells at the division septum in WT cells and the same observation was made in Δ*divIVA* rounded cells (Figure 7A and Table S2). By contrast, while EzrA-GFP localized at midcell in exponentially grown WT cells as well as in Δ*divIVA* and Δ*divIVA*Δ*gpsB* cells, EzrA-GFP formed helical structures that extended across the long axis of the cell in 19.9% of Δ*gpsB* elongated cells (Figure 7B and Table S2) as was found for FtsZ-GFP and DivIVA-GFP (Figures 3A and 6). Western blot control experiments confirmed that GFP-GpsB and EzrA-GFP were produced at similar levels in WT and Δ*divIVA* (and Δ*gpsB* and Δ*divIVA*Δ*gpsB* cells for EzrA) (Figures S8B and S9). These observations are consistent with EzrA serving as a connector between FtsZ and GpsB and/or DivIVA.

### GpsB, but not DivIVA, is required for proper localization and functioning of StkP

**Figure 8:**
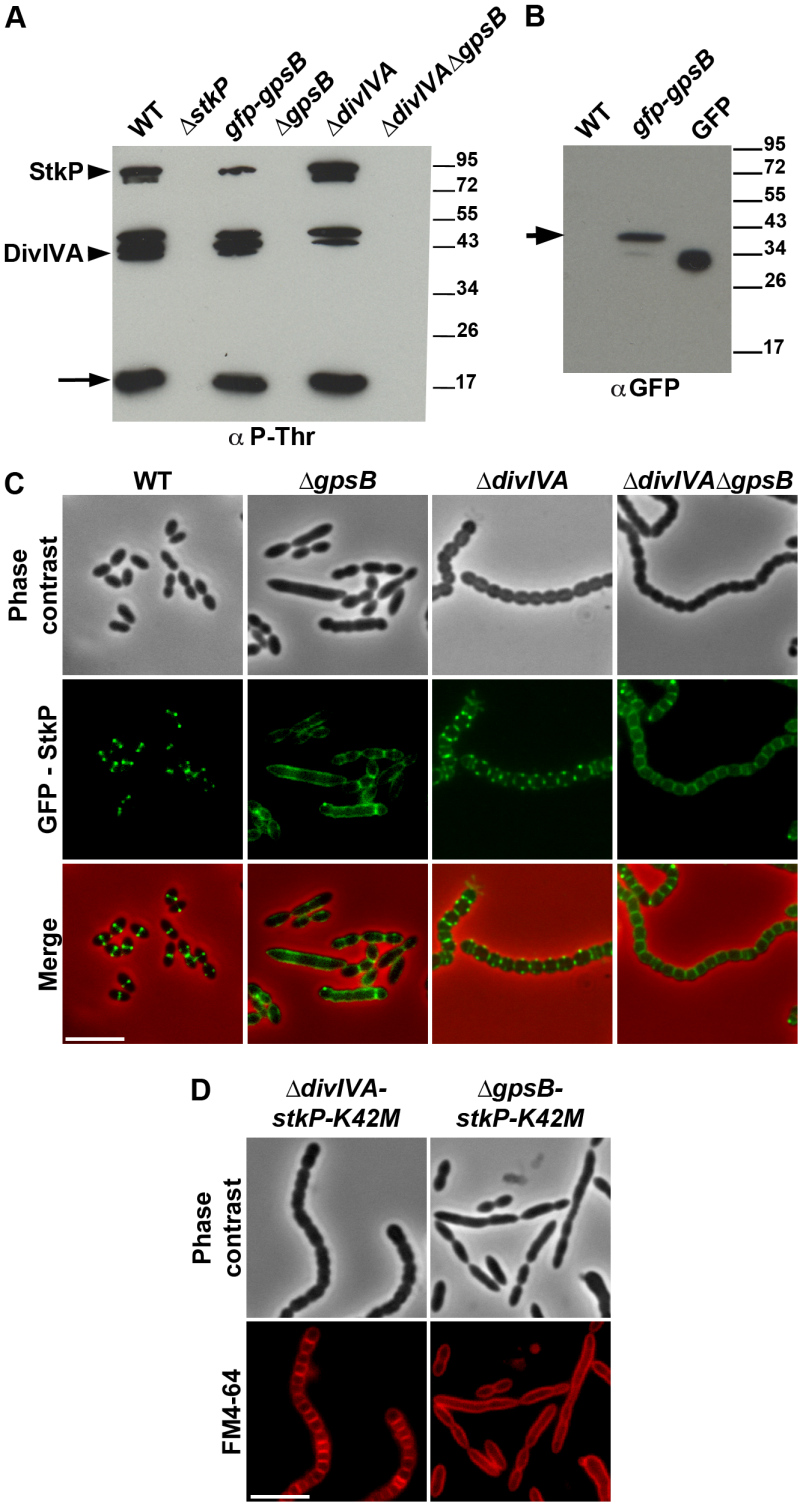
Interplay of GpsB, DivIVA and StkP. (A) Western immunoblot of whole-cell lysates from the wild type (WT), Δ*stkP*, *gpsB::gfp-gpsB*, Δ*gpsB*, Δ*divIVA* and Δ*gpsB*Δ*divIVA* cells grown in THY at 37°C probed with anti-phosphothreonine antibodies. The same amounts (25 µg) of proteins were loaded in all gel lanes. Arrow indicates the signal observed around 15 kDa. The phosphorylation signal for DivIVA and StkP are indicated. (B) Western immunoblot of whole-cell lysates from wild type (WT) or *gpsB::gfp-gpsB* cells probed with anti-GFP antibodies. Purified GFP is used as control. Arrow indicates the signal observed for GFP-GpsB. (C) StkP localization using a GFP N-terminal fusion in WT, Δ*gpsB*, Δ*divIVA* and Δ*gpsB*Δ*divIVA* cells. GFP (green) and phase-contrast (grey) images were taken from a typical field of exponentially grown cells in THY at 37°C. Merged pictures (lower panels) show the overlay of StkP (green) and phase contrast images (red). Scale bar, 5 µm. (D) Cell morphology of *stkP-K42M* cells deficient for DivIVA or GpsB expression. Cells producing a kinase dead-form of StkP (*stkP-K42M*, see [14]) were deleted either for *divIVA* or *gpsB* resulting thus in Δ*divIVA*-*stkP-K42M* and Δ*gpsB*-*stkP-K42M* strains, respectively. Phase contrast microscopy (upper row) and FM4–64 membrane staining (lower row) images of Δ*divIVA*-*stkP-K42M* (left panel) and Δ*gpsB*-stkP-K42M (right panel) exponentially growing cells at 37°C in THY medium. Scale bar, 5 µm.

The elongated phenotype with incomplete septa displayed by Δ*gpsB* cells was reminiscent of that reported for cells expressing the kinase-dead form of StkP and suggested a relationship between these two proteins [14], [15]. Hence, we hypothesized that GpsB could be phosphorylated by StkP in *S. pneumoniae*. The general phosphorylation pattern of crude extracts of pneumococcal cells was thus analyzed using anti-phosphothreonine antibodies. We detected an intense phosphorylation signal around 15 kDa, which could be compatible with the phosphorylation of GpsB (13 kDa) (Figure 8A). Therefore, GpsB from *S. pneumoniae* cells was purified to examine its *in vivo* phosphorylation state using high-resolution based mass spectrometry (Figures S12A–B). No phosphorylated sites were detected suggesting that GpsB is not phosphorylated *in vivo*. To confirm this result, we analyzed the phosphorylation pattern of cells expressing only GFP-GpsB. An intense phosphorylation signal at 15 kDa was still detected and no new phosphorylation signal appeared around 45 kDa, the predicted mass of GFP-GpsB (Figure 8A), though GFP-GpsB was efficiently stained with anti-GFP antibodies (Figure 8B). Altogether, these data show that GpsB is not phosphorylated *in vivo* and that the 15-kDa phosphorylation signal corresponds to another unidentified protein.

In parallel, we analyzed the phosphorylation pattern of Δ*gpsB* cells using anti-phosphothreonine antibodies. Surprisingly, the deletion of *gpsB* abolished not only the phosphorylation of all the substrates of StkP, including DivIVA, but also StkP autophosphorylation itself (Figure 8A). Nevertheless, StkP was expressed at similar levels in WT and Δ*gpsB* cells (Figure S12C). Furthermore, SPR analysis showed that GpsB was able to interact with the inactive cytoplasmic domain of StkP-K42R (K_D_ = 500±60 nM) (Figure S10G). These data raised the question of whether GpsB could affect StkP septal localization [14], [15] (Figure 8C). Therefore, we constructed a Δ*gpsB* mutant harboring a GFP-StkP fusion [14]. Fluorescence microscopy revealed an intense signal distributed all around the cell, consistent with a diffuse membrane localization of GFP-StkP (Figure 8C). StkP localization and general phosphorylation patterns of Δ*divIVA* and Δ*divIVA*Δ*gpsB* cells were also analyzed. While StkP localized to midcell and was able to phosphorylate its targets in the absence of DivIVA, a deficient phosphorylation pattern as well as a StkP diffuse membrane localization were observed in the double mutant (Figures 8A and 8C). In both mutant strains, StkP was produced to similar levels as in WT cells (Figure S12C). These data establish that, although GpsB is not phosphorylated, it is crucial for StkP septal localization and its capacity to autophosphorylate and phosphorylate its substrates, notably DivIVA. By contrast, DivIVA is not required for StkP kinase activity and localization.

### Deletion of *divIVA*, but not of *gpsB* suppresses elongation of *stkP-K42M* cells

Because GpsB is required for StkP septal localization and thus phosphorylation of DivIVA, we investigated the impact of either *gpsB* or *divIVA* deletion on the elongated morphology of cells producing the kinase-dead form of StkP, StkP-K42M [14]. As shown in Figure 8D, Δ*gpsB-stkP-K42M* cells still displayed an elongated phenotype supporting the idea that the main function of GpsB is to allow StkP to phosphorylate its targets. By contrast, the deletion of *divIVA* was found to abrogate *stkP-K42M* cell elongation. Indeed, Δ*divIVA-stkP-K42M* cells formed chains of rounded cells similar to those of the Δ*divIVA*Δ*gpsB* mutant (compare Figure 8D with Figure 1A). This observation confirms that DivIVA is crucial for cell elongation and the late step of cell separation, and further suggests that its non-phosphorylation likely results in aberrant elongation of *stkP-K42M* and Δ*gpsB* cells.

## Discussion

### Roles of DivIVA

According to the current model of PG synthesis in pneumococcus, cell elongation is due to the peripheral PG synthesis. Our observations suggest that peripheral PG is impaired in Δ*divIVA* cells (Figures 1 and S2). In addition, we show that DivIVA co-localizes with FtsZ in Δ*gpsB* elongated cells and that *divIVA* deletion suppresses Δ*gpsB* cell elongation (Figures 1A and 6B). Therefore, we propose that one function of DivIVA is to switch from septal to peripheral PG synthesis to trigger cell elongation. DivIVA performs quite different functions in *B. subtilis* and *Staphylococcus aureus* [27], but it has already been shown to participate in cell wall biosynthesis in bacteria such as *Streptomyces coelicolor*, *Mycobacterium tuberculosis* and *Corynebacterium glutamicum* that either lack or do not require MreB for their vegetative growth. In these bacteria, DivIVA is required for polar growth allowing tip extension and cell elongation [28]–[30]. Pneumococcus is devoid of MreB and any identifiable homologues. Altogether, these observations raise the possibility that DivIVA is crucial for cell elongation in those species in which vegetative growth is not dependent on MreB.

The chaining displayed by Δ*divIVA* cells also suggests that while septum closure leading to the separation of the daughter cell cytoplasms is normal, their final separation is somehow affected, as previously reported for the RX1 strain [6]. DivIVA has been previously found to interact or to contribute to the positioning of some PG hydrolases in the pneumococcus [31], [32] or in autolysin secretion in other bacteria as *Listeria monocytogenes* [33]. The chain phenotype displayed by the Δ*divIVA* mutant is consistent with impairment of PG hydrolysis and remodeling required for final separation of daughter cells.

### Essentiality and role(s) of GpsB

Previous studies using high-throughput gene disruption approaches have suggested that *gpsB* could be essential in pneumococcus [34]–[36]. In this study, we show that GpsB is actually not essential for pneumococcal laboratory strains (Figures 1 and S3). However, and in agreement with the previous observations, no Δ*gpsB* transformants could be obtained with the pathogenic strains D39 and TIGR4 indicating that the requirement for GpsB depends on the genetic background. The recent work of Land and co-workers also suggests that suppressive mutations are required for growth of unencapsulated derivatives of pathogenic strains expressing low level of GpsB [25]. The conditional essentiality of GpsB is reminiscent of the situation with MreC and MreD. These proteins are essential in D39 and TIGR4 pathogenic strains but not in the R6 laboratory strain due to suppressive mutations in PBP1a and in proteins of unknown function in the latter [9].

Inactivation of *gpsB* resulted in severely impaired cell division, with a large fraction of the population appearing as elongated cells with incomplete septa similar to cells producing the kinase-dead form of StkP [14]. This phenotype is accompanied by helical patterns for PG synthesis and FtsZ, along the long axis of the cell (Figures 2 and 3). Interestingly, Z-spiraling was not observed in the work published by Land and co-workers [25]. Rather, the authors detected multiple non-constricted rings of FtsZ in elongated cells. Because GpsB expression was under the control of an inducible fucose promoter, we tentatively attribute the absence of Z-spiraling to low level of *gpsB* expression in absence of fucose, likely preserving Z-ring formation in elongated cells. This hypothesis is consistent with our time-lapse analysis showing Z-spiraling upon cell elongation, eventually splitting to generate several new Z-rings (Figure 3C and Movie S2).

The stimulation of cell elongation and aberrant helical organization of the divisome observed in our study in Δ*gpsB* cells suggest that GpsB is a negative regulator of cell elongation in WT cells. On the other hand, the deletion of *gpsB* has no effect on cell shape and septum closure in the absence of DivIVA (Figure 1A), and septal localizations of PG synthesis, PBP2x, PBP2b, FtsW and RodA are not affected (Figures 2 and 4D). However, the deletion of both *divIVA* and *gpsB* genes has a detrimental effect on cell viability. It could thus be proposed that in absence of DivIVA, GpsB is dispensable for septal PG synthesis but required for optimal cell survival.

We also show that GpsB interacts with StkP and is crucial for both StkP localization at the division site and its ability to phosphorylate its targets, including DivIVA (Figure 8). This represents the first evidence of STPKs regulation by a cell division protein. Consequently, GpsB becomes the major determinant of pneumococcal cell division. Most of StkP targets remain to be identified but their phosphorylation is crucial for cell division [14]. On this basis, one cannot exclude that deficient phosphorylation of StkP targets in the absence of GpsB could favor FtsZ-ring spiraling. In other words, FtsZ could be prone to move in a spiral driving peripheral PG synthesis in the absence of GpsB, yet preventing septal PG synthesis and cell division. The function of GpsB could be more complex than promoting septal PG synthesis during septum closure and also involved in mediating proper condensation of the divisome at midcell.

### Interplay of GpsB, DivIVA and StkP

Analysis of protein-protein interactions revealed that GpsB and DivIVA do not interact with FtsZ but with the cell division EzrA, which itself interacts with FtsZ (Figure S10). Together with the helical organization of EzrA and the co-localization of DivIVA with FtsZ in Δ*gpsB* elongated cells, we propose that septal and peripheral PG synthesis are coordinated with and organized by FtsZ *via* EzrA, GpsB and DivIVA.

Considering the opposing function of DivIVA and GpsB in cell elongation, and the finding that inactivation of *divIVA* in Δ*gpsB* cells results in the disappearance of elongated cells, we propose that GpsB is required to confine PG synthesis at the division site and to negatively control cell elongation promoted by DivIVA. GpsB and DivIVA are also found to interact. Therefore, we propose that GpsB and DivIVA constitute a molecular switch, connected to FtsZ *via* EzrA, that orchestrates the production of peripheral (cell elongation) and septal (cell division) PG to confer to the pneumococcus its characteristic ovoid shape. How could this switch operate? The finding that inactivation of *gpsB* affects both StkP septal localization and kinase activity, and thus DivIVA phosphorylation, leads us to propose that cell elongation is stimulated by non-phosphorylated DivIVA and that DivIVA phosphorylation by StkP abolishes its ability to promote cell elongation. Suppression of the elongated cell shape of the *stkP-K42M* mutant upon *divIVA* deletion is consistent with this hypothesis (Figure 8D).

### Toward a single PG synthesis machine?

The current model of PG synthesis in *S. pneumoniae*, and more generally in ovococci, proposes that the two modes of PG synthesis depend on the action of two distinct machineries [12], as described for rod-shaped bacteria. Recently, Land and co-workers have analyzed the localization of PBP2x and PBP1a over the cell cycle [25]. These two enzymes display similar localization patterns in pre- and mid-divisional cells, but not during septum closure. Indeed, PBP1a localized as a ring larger than that of PBP2x. This observation was interpreted as supporting the existence of two distinct PG synthesis machineries. While PBP2x is essential and participates in cell constriction (septal PG synthesis), the role of PBP1a in PG synthesis remains elusive. A *pbp1a* mutant is affected both in length and width but cells grow normally with no viability defects and cells remain ovoid rather than being elongated or rounded [9], [37], [38]. In the two-machinery model, PBP1a would be involved in both elongation and constriction. That PBP2x and PBP1a display different localization dynamics during septum closure does not necessarily imply that they belong to two distinct machineries. Here we have analyzed the localization of PBP2x and FtsW as well as PBP2b and RodA, which are proposed to be specific for septal and peripheral PG synthesis, respectively, in the two-machinery model. We show that they all co-localize with helix-shaped FtsZ in elongated Δ*gpsB* cells (Figure 5B). In addition, we failed to delete the genes encoding PBP2x, PBP2b, FtsW or RodA in Δ*gpsB*, Δ*divIVA* or Δ*divIVA*Δ*gpsB* cells indicating that all these proteins remain essential even when septal or peripheral PG synthesis is impaired. Therefore, our data hardly fit with (and challenge) the two-machinery model. An exciting and promising alternative conciliating the data reported by Land and co-workers with ours would be that the four proteins are present in a same unique complex, ensuring both septal and peripheral PG synthesis, whose composition varies in the course of the cell cycle. A previous study has demonstrated that a first short step is dedicated to cell elongation (around 300 nm) (peripheral PG synthesis) [39]. This is followed by a second step, in which cell constriction (septal PG synthesis) occurs simultaneously with elongation at mid-cell of the forming daughter cells, and a third step dedicated to constriction. In the second step, PG synthesis is distributed along progressively constricting circles converging toward the future new cell pole to achieve both elongation and septation. These observations are consistent with a finely tuned single machinery allowing concomitant cell elongation and constriction, with components displaying different localization dynamics toward the future equatorial division site. Considering these constraints imposed by an ovoid cell shape, a unique machinery thus represents an attractive mean to achieve PG synthesis along progressively constricting circles. Deciphering these mechanistic questions will certainly require implementing higher resolution microscopy approaches than 3D-SIM, such as PALM or STORM, to assess the dynamics of each components of the division machinery over the pneumococcus cell cycle and particularly during the second step involving simultaneous cell constriction and elongation.

**Figure 9:**
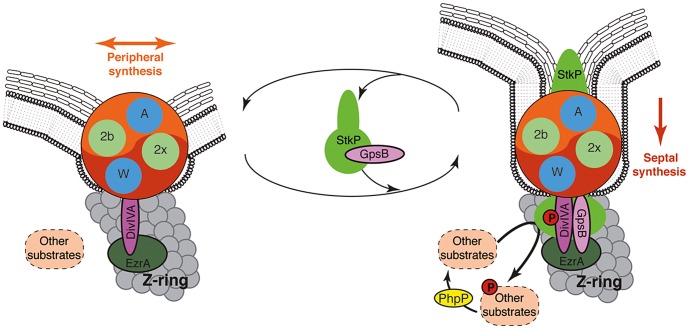
Models for PG synthesis in *S. pneumoniae*. In this model, a large membrane PG assembly complex (Yin Yang circle) contains both the septal (red) and the peripheral (orange) PG assembly machineries. The two transpeptidases PBP2x and PBP2b (noted 2x and 2b) and the two lipid-flippases FtsW and RodA (noted W and A) are indicated in green and blue, respectively. Non-phosphorylated forms of DivIVA and other StkP substrates are required for cell elongation and thus peripheral PG synthesis. GpsB is not *per se* involved in the production of the cross-wall, but is required at the septum to localize StkP (light green oval), to allow the phosphorylation of StkP substrates including DivIVA and to favor production of septal PG by down-regulating peripheral PG synthesis. The paralogs GpsB (pink oval) and DivIVA (purple oval) constitute a molecular switch that connects, together with EzrA (green oval), the Z-ring with the PG assembly complex. StkP kinase activity, counterbalanced by the phosphatase PhpP (yellow oval) [15] and triggered by GpsB, modulates the function of a set of proteins (dashed ovals) including DivIVA [14]. The StkP/DivIVA/GpsB triad is thus proposed to orchestrate and to finely tune production of septal and peripheral peptidoglycan synthesis responsible for the ovoid-shape of pneumococcus.

Using a depletion approach, Berg and co-workers recently reported that lowering the amount of either PBP2b or PBP2x in pneumococcus results in lentil-shaped and lemon-shaped-cells, respectively [40]. These cell shapes are distinct from that of Δ*divIVA* rounded cells and Δ*gpsB* elongated cells. An interpretation could be that while the catalytic activity of the PBPs is important to specifically achieve septal or peripheral synthesis, they are both structurally (physically) required for the two PG synthesis modes. This would be consistent with our observations and further supports our model in which PG synthesis would depend on a single machine responsible for both septal and peripheral PG synthesis (Figure 9). In such a model, we propose that the StkP/DivIVA/GpsB triad finely tunes this machine to dictate the type of PG (septal or peripheral) produced. Investigating the underlying regulatory mechanism, which might involve modification of DivIVA interactions with EzrA, GpsB, or other partners in the divisome presumably *via* StkP-driven phosphorylation, will likely improve the understanding of how septal and peripheral PG synthesis are coordinated.

### The StkP/DivIVA/GpsB triad: A regulatory device conserved in other bacterial species?

**Figure 10:**
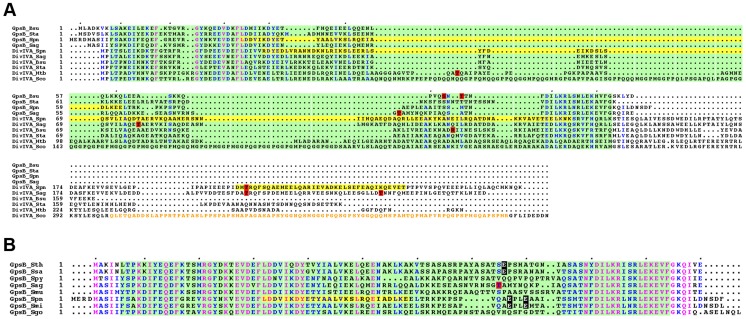
Alignment for GpsB and DivIVA proteins from several bacteria. (A) Multiple sequence alignments of GpsB and DivIVA sequences from streptococci and Gram-positive bacteria. Protein sequences similar to that of pneumococcus GpsB and DivIVA were identified by BLAST searches and aligned using CLUSTALW. Spn: *S. pneumoniae*; Sag: *S. agalactiae*, Bsu: *B. subtilis*; Sta: *S. aureus*, Mtb: *M. tuberculosis*; Sco: *S. coelicolor*. Yellow highlights the potential coiled-coil motifs retrieved from UniProtKB/Swiss-Prot:Q8CWP9 and UniProtKB/Swiss-Prot:C1CIN3 entry annotations for Spn-DivIVA (residues 34–135 and 199–236) and Spn-GpsB (36–63) respectively. The PF05103 PFAM DivIVA family signatures are mapped as green open boxes for DivIVA and GpsB. When identified, phosphorylation sites are red boxed. The *S. coelicolor* DivIVA phosphopeptide containing unidentified phosphorylation sites are highlighted in orange letters. Identical residues are in pink letters and positions showing conservation of similar residues are in blue. Dots indicate gaps introduced in sequences during alignment computation. The figure was rendered with the ESPript server [54]. (B) Multiple sequence alignments of GpsB sequences from streptococci. Protein sequences were aligned using CLUSTALW. Spy: *S. pyogenes*; Sag: *S. agalactiae*, Smu: *S. mutans*; Sth: *S. thermophylus*; Ssa: *S. salivarius*; Spn: *S. pneumoniae*; Smi: *S. mitis*, Sgo: *S. gordonii*. The PFAM PF05103 DivIVA family signatures are mapped as green boxes. Yellow highlights the potential coiled-coil motifs retrieved from UniProtKB/Swiss-Prot:C1CIN3 entry annotations for Spn-GpsB (36–63). The phosphothreonine identified for *S. agalactiae* GpsB is red boxed. Glutamic acids possibly mimicking threonine phosphorylation are black boxed with white letters. Identical residues are in pink letters and positions showing conservation of similar residues are in blue. Dots indicate gaps introduced in sequences during alignment computation. The figure was rendered with the ESPript server [54].

Phosphorylation of GpsB and/or DivIVA homologs has previously been detected in *B. subtilis*, *S. coelicolor*, *S. agalactiae* and *M. tuberculosis* [20], [21], [41], [42]. However, phosphorylation sites are not conserved or occur in regions of poor amino acid conservation (Figure 10A). In addition, phosphothreonines can be replaced by glutamic acids, as revealed by alignment of GpsB sequences from several streptococci (Figure 10B). Interestingly, negatively charged amino acids (Asp/Glu) can mimic the phosphorylated state of a protein. A recent comparative genomic study indicated that nature uses this trick in reverse by evolving serine, threonine, and tyrosine phosphorylation sites from Asp/Glu residues [43]. It is thus possible that GpsB and DivIVA phosphorylation by StkP is a widespread means for finely tuning cell-wall synthesis and defining bacterial cell shape though the underlying mechanism may differ between species.

## Materials and Methods

### Strains, plasmids, primers and growth conditions

For growth experiments, *S. pneumoniae* strains were cultivated at 37°C in Todd-Hewitt Yeast (THY) broth (Difco). For induction of P_Zn_, ZnCl_2_ was added at the concentration of 0.15 mM. For construction of *S. pneumoniae* mutants, transformation was performed as described previously [44], using precompetent cells treated at 37°C with synthetic competence stimulating peptide 1 (CSP 1) to induce competence. Transformants were plated into THY-agar supplemented with 3% (vol/vol) defibrinated horse blood and then incubated for 120 min at 37°C. Selection was then performed by adding a 10 ml THY-agar overlay containing the appropriate antibiotic (streptomycin 200 µg/ml, kanamycin 250 µg/ml, tetracyclin 2,5 µg/ml) and overnight incubation at 37°C. For viability assays, several samples of exponentially growing cells were taken every 30 min, diluted appropriately and plated onto THY-agar supplemented with horse blood. After overnight incubation, colony-forming units (CFU) were counted and the percentage of viability of mutant strains was expressed relatively to the WT strain. The *Escherichia coli* XL1-Blue strain was used as a host for cloning. *E. coli* BL21(DE3) strain was used as host for overexpression. The *E. coli* BTH101 was used as host for bacterial two-hybrid analysis. Luria–Bertani (LB) broth and agar supplemented with appropriate antibiotic (tetracyclin 15 µg/ml, ampicillin 100 µg/ml, and kanamycin 25 µg/ml) were used for routine growth at 37°C. The nucleotide sequences of all synthesized DNA fragments were checked to ensure error-free amplification. Strains used in this study are listed in Table S3.

### Construction of plasmids

DNA fragments coding for GpsB, DivIVA, FtsZ, inactive StkP cytoplasmic domain and EzrA without its N-terminal transmembrane domain were obtained by PCR using chromosomal DNA from *S. pneumoniae* R800 strain as template and oligonucleotides described in Table S4, section 2. Site directed mutagenesis of StkP kinase domain was achieved by 2 successive PCRs using chromosomal DNA as template and primer pair IX/XI and then the resulting DNA fragment and primer X. The obtained DNA fragments were cloned between the *Nde*I and *BamH*I cloning sites of the pETPhos plasmid (except for *ezrA* that has been inserted using *Nhe*I and *BamH*I) [45]. To construct P_Zn_-*gfp-gpsB*, *gfp* was first amplified using the primer pair XII/XIII (Table S4 section 3) using pUC57-*gfp* as template [46]. After digestion with *Age*I and *Not*I, *gfp* was cloned into pCM38 (gift form C. Morlot, IBS, Grenoble) previously opened with the same enzymes resulting in P_Zn_-*gfp*. pCM38 is a modified version of pJWV25 [47] in which an *Age*I restriction site has been inserted upstream of the *gfp+* gene. Then, *gpsB* was amplified using the primer pair XIV/XV (Table S4 section 3) using pneumococcus WT chromosomal DNA. The amplified fragment was then digested by *Spe*I and *Not*I and inserted in P_Zn_-*gfp* resulting in P_Zn_-*gfp-gpsB.* To construct plasmids for bacterial two-hybrid, DNA fragments were amplified by PCR using specific primers pairs presented in Table S4, section 4. The PCR DNA fragments were then digested by *Acc*65I and *Xba*I and ligated into either pKNT25 or pUT18 vectors [26]. The nucleotide sequences of all final PCR DNA fragments were checked to ensure error-free amplification. Plasmids and primers used in this study are listed in Tables S3 and S4, respectively.

### Allelic replacement mutagenesis

*S. pneumoniae* strains were constructed by transformation in R800 and are therefore isogenic. We used a two-step procedure, based on a bicistronic *kan-rpsL* cassette called Janus [48] to delete, or replace the genes of interest by their *gfp* or *rfp* fusion forms. This procedure avoids polar effects and allows a physiological level of expression of GFP and RFP fusions. An exhaustive description of the procedure is provided in Supplemental Materials and Methods (Text S1). The genes encoding GFP and RFP were from [46] and [15], respectively.

### Protein purification

Recombinant plasmids overproducing GpsB, FtsZ EzrA, DivVA and inactive StkP cytoplasmic domain (StkP-K42R) were transformed into the BL21(DE3) *E. coli* strain. The transformants were grown at 37°C until the culture reached an OD_600_ = 0.4. Expression was induced by adding IPTG to a final concentration of 0.5 mM and incubation was continued for 3 h. Proteins were extracted, purified on a Ni-NTA agarose column (Qiagen) and dialyzed overnight at 4°C as previously described [14]. The concentration of protein was determined using a Coomassie Assay Protein Dosage Reagent (Uptima) and aliquots were stored at −80°C.

To purify GpsB from *S. pneumoniae* cells, we constructed a strain in which *gpsB* is fused to a DNA fragment encoding for 6 histidines at the chromosomal locus. We checked that cells grew as the WT cells and displayed proper cell shape. This strain was cultured in THY medium at 37°C until OD_550_ reached 0.4. After centrifugation, the pellet was suspended in buffer A (50 mM Tris-HCl pH7.5, 10% (v/v) glycerol, 200 mM NaCl, 10 mM imidazole, 0.3% (w/v) SDS) supplied with 1 mg/L lysosyme, 6 mg/L DNase/RNase, 1× cocktail of anti-protease (Roche) and 0.1% (v/v) anti-phosphatase (Sigma). The cells were then incubated at 4°C for 10 min and opened by sonication. The lysate was supplied with 1% (v/v) Triton X-100 and further incubated at 4°C for 15 min. Then, the lysate was subjected to ultracentrifugation of 14,000× g for 30 min. Ni-NTA agarose was equilibrated with buffer A′ (50 mM Tris-HCl pH7.5, 10% (v/v) glycerol, 200 mM NaCl) and then incubated with the ultracentrifuged supernatant. The resin was washed twice with buffer A supplied with 0.1% (v/v) Triton X-100 and then twice with buffer B (buffer A′ containing 20 mM imidazole and 0.1% (v/v) Triton-X100). Elution was carried out with buffer C (buffer A containing 300 mM imidazole and 0.1% (v/v) Triton-X100). Eluted fractions were collected and added with 0.02% (w/v) deoxycholate and 8% (w/v) trichloroacetic acid and shake vigorously. After centrifugation of 13,200× g at 4°C for 30 min, the supernatant was discarded and the pellet was resuspended in SDS-PAGE loading buffer. pH was adjusted using 1.5 M Tris-HCl pH8.8. The resulting samples were separated by 15% SDS-PAGE after boiling for 5 min.

### Microscopy techniques

TEM, SEM, fluorescence and immunofluorescence microscopy were carried out as previously described [14]. Cells were grown at 37°C in THY broth and analyzed when the OD reached Abs_550_ = 0.1 Polyclonal antibody specific for FtsZ [49] was used at 1/200. Slides were visualized with a Zeiss AxioObserver Z1 microscope fitted with an Orca-R2 C10600 charge-coupled device (CCD) camera (Hamamatsu) with a 100× NA 1.46 objective. Images were collected with AxioVision (Carl Zeiss) and analyzed with ImageJ (http://rsb.info.nih.gov/ij/). For TEM, cells were examined with a Philips CM120 transmission electron microscope equipped with a Gatan Orius SC200 CCD camera. For SEM, cells were observed with a Quanta 250 FEG (FEI) scanning electron microscope. For PG labeling with Bodipy-FL-amino-D-alanine (BADA), the procedure used was adapted from [23], [24]. Exponentially growing cells of (OD_550_ = 0.1) were incubated for 4 min at 37°C with 500 µM of BADA. Cells were then washed three times with Phosphate Buffer Saline (PBS) pH 7.4. Then, 0.7 µl of the mixture was placed on slides and observed under the microscope. Time-lapse microscopy was performed as described [50] using an automated inverted epifluorescence microscope Nikon Ti-E/B equipped with the perfect focus system (PFS, Nikon) and a phase contrast objective (CFI Plan Fluor DLL 100× oil NA1.3), a Semrock filter set for GFP (Ex : 482BP35; DM : 506; Em : 536BP40), a Nikon Intensilight 130W High-Pressure Mercury Lamp, a monochrome OrcaR2 digital CCD camera (Hamamatsu) and an ImagEM-1K EMCCD camera (Hamamatsu). Briefly, after gentle thawing of THY stock cultures, aliquots were inoculated at OD_550_ = 0.006 in C+Y medium and grown at 37°C to an OD_550_ of 0.3. These precultures were inoculated (1/100) in C+Y medium and incubated at 37°C to an OD_550_ of 0.1 unless otherwise specified. Two microliters were directly spotted on a microscope slide containing a slab of 1.2% C+Y agarose. The microscope is equipped with a chamber thermostated at 30°C. Images were captured every 5 minutes and processed using Nis-Elements AR software (Nikon). All fluorescence images were acquired with a minimal exposure time (exposure time: 2 seconds; camera gain: 50; light attenuation with neutral-density filters: 25%) to minimize bleaching and phototoxicity effects. GFP fluorescence images were false colored green and overlaid on phase contrast images.

### Immunoblot analysis

Detection of *in vivo* phosphorylated proteins in crude extracts of *S. pneumoniae* strains was performed after SDS-PAGE by immunoblotting using an anti-phosphothreonine polyclonal antibody (Cell Signaling) at 1/2000 as described in [14]. A goat anti-rabbit secondary antibody HRP conjugate (Biorad) was used at 1/5000. Detection of StkP and GFP fusions were performed using a rabbit polyclonal antibody specific for StkP [14] and rabbit anti-GFP (AMS Biotechnology).

### Mass spectrometry

To examine GpsB *in vivo* phosphorylation, GpsB was analyzed by SDS-PAGE after purification (see Protein purification). An in gel digest using trypsin was performed, followed by a phosphorylated peptide enrichment procedure with TiO2 beads as previously described [51], with minor modifications: TiO_2_ beads (10 µm) (MZ Analysetechnik, Mainz, Germany) were incubated with 2,5 dihydrobenzoic acid in 80% acetonitrile (final concentration 30 g/L) prior to phosphopeptide enrichment. 5 mg of TiO_2_ beads were added to the sample and incubated at room temperature on a rotating carousel for 30 minutes. After washing in 1 mL 30% acetonitrile/3% TFA and 80% acetonitrile/0.1% TFA for 10 min each, the phosphopeptides were eluted from the TiO_2_ spheres with 3×100 µL of 40% ammonium hydroxide solution in 60% acetonitrile, pH 10.5. The sample volume was reduced in a vacuum centrifuge at room temperature and brought to a final volume of 6 µL for nano-LC-MS/MS analysis. NanoLC-MS/MS-experiments were performed on an EASY-nLCt system (Proxeon Biosystems,) connected to an LTQ-Orbitrap XL or Elite. For proteome analysis, peptides were applied onto a 15 cm nano-HPLC column, in-house packed with reverse-phase 3 µm C18 spheres (Dr. Maisch, Ammerbuch, Germany) at a flow rate of 500 nL/min in 0.5% acetic acid. The peptides were eluted using a segmented 90 min gradient of 5–33% of Solvent B (80% acetonitrile in 0.5% acetic acid) at a constant flow rate of 200 nL/min. Peptide were ionized via the electrospray ion source (ESI) (Proxeon Biosystems, Odense, Denmark). The mass spectrometer was operated in the positive ion mode with the following acquisition cycle: one initial full scan in the Orbitrap analyzer (MS) was followed by fragmentation through rapid collision induced dissociation (CID) of the 20 most intense multiply charged precursor ions in the linear ion trap analyzer (LTQ). The full scan was performed range of m/z 300–2,000 at a resolution of 120,000 (defined at m/z = 400). Target values were set at 1E6 and 5E3 charges for MS or MS/MS, respectively. Sequenced precursor ions were subjected to dynamic exclusion (set for 90 seconds). The LTQ Orbitrap XL was used for the detection of phosphorylation sites in the same way as above but with slight modifications: CID was performed on the 5 most intense precursor ions. Multi stage activation (MSA) was applied in all MS/MS events when a neutral loss event was detected on the precursor ions depending on their charge state: singly (−97.97 Th), doubly (−48.99 Th) and triply (−32.66 Th).The full scan was set at 60,000 and the lock-mass option [52] was enabled for real time recalibration of MS spectra. All RAW files were processed with the MaxQuant software version 1.2.2.9 [53]. N-acetylation of protein (N term+42.010565 Da), N-pyro-glutamine (Gln _17.026549), oxidized methionine (+15.994915 Da) and phosphorylation of serine, threonine and tyrosine (Ser/Thr/Tyr +79.966331 Da) were searched as variable modifications. The database used to search all submitted peak lists was uniprot *S.pneumoniae* ATCC BAA-255 R6.

### Analysis of protein-protein interactions

Bacterial two-hybrid experiments were performed according to the manufacturer's instructions (Euromedex). The picture was taken after 40 h of growth at 30°C onto LB-agar plates containing X-gal (40 µg/ml), 0.5 mM IPTG and appropriated antibiotics. For analyses using surface plasmon resonance (SPR), real time binding experiments were performed on a BIAcore T100 biosensor system (GE Healthcare). EzrA or GpsB (ligand) were covalently coupled through their amino groups to the surface of a CM5 sensorchip according to the manufacturer's instructions. Increasing concentrations (0.002, 0.005, 0.1, 0.2, 0.5 and 1 µM from bottom to top) of DivIVA, EzrA, StkP, GpsB, or FtsZ (analyte) were injected over the surface of the sensorchip at a flow rate of 30 µL/min in 10 mM HEPES pH 7.4, 150 mM NaCl, 0,005% surfactant. For all experiments, aspecific binding to the surface of the sensorchip was substracted by injection of the analytes over a mocked derivatized sensorchip. The resulting sensorgrams were analyzed using BIAevaluation software (GE Healthcare). K_D_ values were calculated from the equilibrium resonance signal (Req) as a function of the analyte concentration. Req values were estimated by extrapolation to infinite time using plots of resonance signal as a function of the reciprocal of time. Apparent K_D_ were then calculated by nonlinear fitting to the expression Req = RmaxC/(KD+C), where Rmax is the maximum binding capacity of the surface and C is the analyte concentration. The goodness of the fit was assessed by inspecting the *χ* ^2^ values. The measurements were made in triplicate.

## Supporting Information

Figure S1 Cell morphology and growth of Δ*divIVA* and Δ*gpsB* mutants repaired back to WT. *divIVA* and *gpsB* genes were inserted back to their genuine chromosomal locus in either the Δ*divIVA* mutant or the Δ*gpsB* mutant to obtain *divIVA* ^+^ and *gpsB* ^+^ strains, respectively. (A) Cell shape of *divIVA* ^+^ and *gpsB* ^+^ cells. Phase contrast microscopy (left panel) and FM4–64 membrane staining (right panel) images of exponentially growing cells at 37°C in THY medium. Scale bar, 5 µm. (B) Growth of *divIVA* ^+^ and *gpsB* ^+^ cells compared to WT cells. Strains were grown in THY medium at 37°C in a JASCO V-630 Biospectrophotometer. The OD_550_ was read automatically every 10 min.
(TIF)

Figure S2 Morphology and cell length of Δ*divIVA* cells. (A) Δ*divIVA* cells were grown at 37°C in THY medium and observed by scanning electron microscopy. Scale bar, 10 µm. (B) Frequency of the length parameter of Δ*divIVA* cells compared to WT cells. Strains were grown in THY medium at 37°C up to OD_550_ = 0.1. The lengths of at least 500 cells of WT and Δ*divIVA* strains, based on phase-contrast images, were measured using ImageJ.
(TIF)

Figure S3 GpsB is required for *S. pneumoniae* growth and cell division. (A) Effect of *gpsB* deletion on pneumococcal growth. WT (black curve) and Δ*gpsB* (red curve) strains were grown in THY medium at 37°C. The OD_550_ was read automatically every 10 min. (B) Frequency of the length parameter of Δ*gpsB* cells compared to WT cells. Strains were grown in THY medium at 37°C up to OD_550_ = 0.1. The lengths of at least 500 cells of WT and Δ*gpsB* cells, based on phase-contrast images, were measured using ImageJ. (C) Phase contrast microscopy (grey) and FM4–64 membrane staining (red) of *gpsB*-deficient (upper row) and WT (lower row) R6 and RX1 growing cells at 37°C in THY medium. Scale bar, 5 µm.
(TIF)

Figure S4 Analysis of WT cells expressing FtsZ-GFP. (A) Growth curves of WT strains expressing either FtsZ (black) or FtsZ-GFP (red) as the only source of FtsZ from its endogenous chromosomal locus grown in THY medium at 37°C. The OD_550_ was read automatically every 10 min. (B) Expression of the FtsZ-GFP fusion in WT and Δ*gpsB* cells. Cells were grown in THY medium at 37°C to OD_550_ = 0.3. Crude extracts (25 µg) of WT or Δ*gpsB* cells expressing FtsZ fused to GFP were analyzed by SDS-PAGE, electro-blotted onto a PVDF membrane and probed with anti-GFP antibodies. Purified GFP and a crude extract of WT cells not producing FtsZ-GFP were used as controls.
(TIF)

Figure S5 FtsZ localization in Δ*gpsB* cells. Same image as in Figure 3A but unprocessed. Arrows show cells without FtsZ-GFP signal in Figure 3A. Phase contrast (left), GFP fluorescent signal (middle) and overlays (right) between phase contrast (red) and GFP (green) images are shown. Scale bar, 5 µm.
(TIF)

Figure S6 Growth curves of WT cells expressing GFP-PBP2x, GFP-PBP2b, FtsW-GFP or RodA-GFP fusions. (A) Growth curves of WT cells (black) and cells expressing either GFP-PBP2x (orange) or GFP-PBP2b (purple) (left panel), or FtsW-GFP (red) or RodA-GFP (blue) (right panel) in THY medium at 37°C. The OD_550_ was read automatically every 10 min. (B) Same as above but in cells also expressing the FtsZ-RFP fusion. All fusion proteins are the only source of PBP2x, PBP2b, FtsW, RodA or FtsZ in the cells. The fusion genes encoding these proteins substitute the corresponding native genes at their chromosomal locus.
(TIF)

Figure S7 Expression of GFP-PBP2x, GFP-PBP2b, FtsW-GFP or RodA-GFP fusions. Expression of GFP-PBP2x and GFP-PBP2b fusions (upper row) and FtsW-GFP and RodA-GFP fusions (lower row) in WT, Δ*gpsB*, Δ*divIVA* and Δ*divIVA*Δ*gpsB* strains. Cells were grown in THY medium at 37°C. Crude extracts (25 µg) were analyzed by SDS-PAGE, electro-blotted onto a PVDF membrane and probed with anti-GFP antibodies.
(TIF)

Figure S8 Growth curves and expression of DivIVA-GFP and EzrA-GFP fusions. (A) Growth curves of WT cells (black) and cells expressing either DivIVA-GFP (red) or EzrA-GFP (green) in THY medium at 37°C. The OD_550_ was read automatically every 10 min. DivIVA-GFP and EzrA-GFP were produced as the only source of DivIVA and EzrA. (B) Expression of EzrA-GFP and DivIVA-GFP fusions in WT and Δ*gpsB* strains. Cells were grown in THY medium at 37°C to OD_550_ = 0.3. Crude extracts (25 µg) of WT or Δ*gpsB* cells expressing either DivIVA or EzrA fused to GFP were analyzed by SDS-PAGE, electro-blotted onto a PVDF membrane and probed with anti-GFP antibodies.
(TIF)

Figure S9 Expression of GFP fusions in Δ*divIVA* and Δ*divIVA*Δ*gpsB* cells. (A) Expression of GFP-fused FtsZ and EzrA expressed as a single copy substituting the chromosomal *ftsZ* and *ezrA* genes, respectively, in Δ*divIVA* strain. For GpsB, expression from the P_Zn_ promoter was assessed both in WT and Δ*divIVA* strains. Crude extracts (25 µg) of WT or Δ*gpsB* cells expressing FtsZ fused to GFP were analyzed by SDS-PAGE, electro-blotted onto a PVDF membrane and probed with anti-GFP antibodies. A crude extract of WT untagged cells was used as control. (B) Same as above for FtsZ and EzrA GFP fusions but in Δ*divIVA*Δ*gpsB* cells.
(TIF)

Figure S10 Analyses of the interactions. (A) Bacterial two-hybrid analyses. Plasmids expressing either the T18 or the T25 fragments of the adenylate cyclase protein fused to the C-terminus of DivIVA, GpsB, FtsZ and EzrA were constructed and the interactions between two candidates were assessed after co-transformation of T18- and T25-constructs in *E. coli* BTH101 and growth for 40 h on LB/X-Gal/IPTG plates. The blue coloration indicates positive interactions. (B) Purification of GpsB, EzrA, DivIVA, FtsZ and StkP-K42R cytoplasmic domain. Proteins were overproduced in *E. coli* BL21 as 6his-tagged fusion proteins. After purification using a Ni-NTA resin, purified proteins were analyzed by SDS-PAGE. (C–G) SPR analyses of interactions. (C–G, left panels) Kinetics of the interactions by Plasmon Surface Resonance (SPR) of EzrA, GpsB, DivIVA, FtsZ and StkP-K42R cytoplasmic domain. EzrA or GpsB were covalently coupled through their amino groups to the surface of a CM5 sensorchip. Increasing amounts of either GpsB (C) DivIVA (D) or FtsZ (E) were injected onto the EzrA-coupled sensorship. Similarly, increasing amounts of either DivIVA (F) or StkP (G) were injected onto the GpsB-coupled sensorship. RU: resonance units. The measurements were made in triplicate. (C–G, right panels) Non-linear regression fits to the equilibrium resonance signal (Req), obtained by extrapolation to infinite time *vs.* analyte concentration, used to obtain apparent equilibrium dissociation constant (KD) (see Materials and Methods).
(TIF)

Figure S11 Localization of GFP fused to GpsB expressed as a single copy in WT cells. WT cells expressing either a N-terminal GFP-GpsB (upper row) or a C-terminal GpsB-GFP (lower row) fusion as a single copy substituting the chromosomal *gpsB* gene were grown in THY medium at 37°C. Scale bar, 5 µm.
(TIF)

Figure S12 Phosphorylation of GpsB and StkP. (A) and (B) Analysis of GpsB *in vivo* phosphorylation in WT cells. After purification from WT cells, GpsB was analyzed by mass spectrometry (see Materials and Methods). (A) Overview into the coverage of GpsB. 93% of the amino acid sequence is identified. (B) Peptides identified are marked in yellow. MS/MS identified peptides of GpsB, along with the PEP and Andromeda scores (FDR 1%). The PEP score (Posterior probability score) represents the probability of a false hit based on the length of the peptide and the identification score that the peptide received. The smaller the PEP score, the higher the statistical probability is that the peptide was correctly identified. Searches were performed at an FDR threshold level of 1%. A search where no FDR threshold was also applied in order to ensure that no phosphorylation sites were filtered out. Peptides were identified with no phosphorylation sites detected. (C) Western immunoblot of whole-cell lysates from WT, Δ*gpsB*, Δ*divIVA* and Δ*gpsB*Δ*divIVA* cells grown in THY at 37°C probed with anti-StkP-PASTA antibodies [14]. The same amounts (25 µg) of cell crude extracts have been loaded in all gel lanes. Arrow indicates the expression signal detected for StkP.
(TIF)

Table S1 Statistical analysis of WT, Δ*gpsB*, Δ*divIVA* and Δ*gpsB*Δ*divIVA* mutant cell parameters. Average area, perimeter, length, AR (major axis/minor axis ratio) +/− SD and chaining of exponentially growing cells. The number of counted cells is in parentheses.
(XLSX)

Table S2 Statistical analysis of GFP-fused protein localization patterns and BADA staining in WT, Δ*gpsB*, Δ*divIVA* and Δ*gpsB*Δ*divIVA* mutant cells. The number of counted cells is in parentheses.
(XLSX)

Table S3 Strains and plasmids.
(XLSX)

Table S4 List of primers.
(XLSX)

Text S1 Supplemental Materials and Methods. Exhaustive description of the procedure used for allelic replacement mutagenesis and construction of mutant strains.
(PDF)

Video S1 Deconvolution microscopy of Δ*gpsB* cells producing FtsZ-GFP. Δ*gpsB* cells producing FtsZ-GFP (green) grown in THY medium at 37°C were stained with FM4–64 (red) and subjected to deconvolution microscopy. 20 images of optical sections of fluorescence from GFP (and FM4–64 when applicable) were collected at spacings of 0.2 µm. Images were deconvoluted through 30 iterations using the Huygens deconvolution software. The deconvoluted images were then projected using Imaris software. Scale bar, 2 µm.
(AVI)

Video S2 Time-lapse analysis of Δ*gpsB* cells producing FtsZ-GFP. The movie shows an overlay of GFP (green) and phase-contrast (gray) images. Scale bar, 2 µm).
(AVI)
