# Supplementary figures and images for: Interplay of the Serine/Threonine-Kinase StkP and the Paralogs DivIVA and GpsB in Pneumococcal Cell Elongation and Division
Source: PLoS Genet. 2014 Apr 10;10(4):e1004275. doi: 10.1371/journal.pgen.1004275 (PMC3983041; doi:10.1371/journal.pgen.1004275)

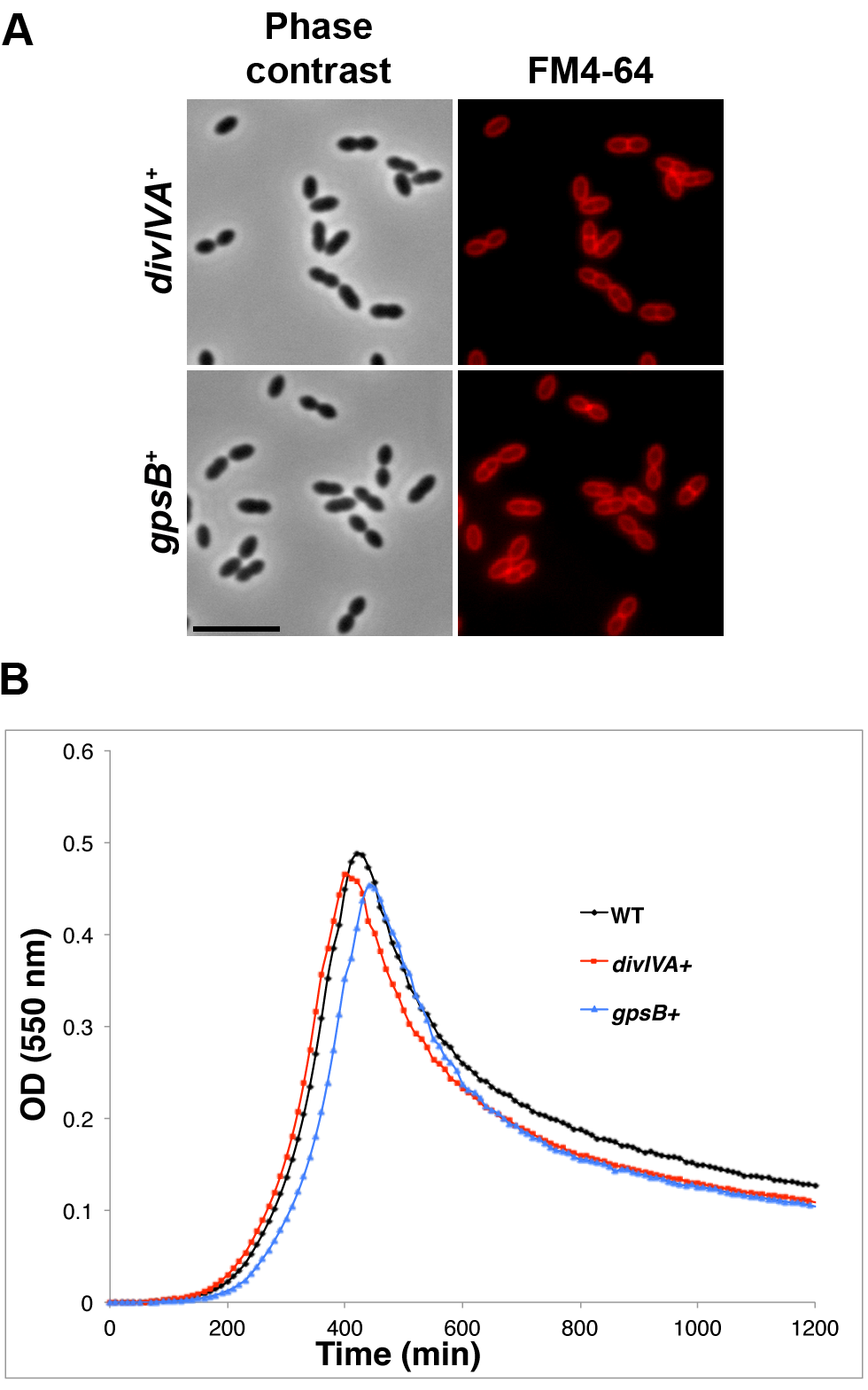

Supplement: Figure S1 — Cell morphology and growth of ΔdivIVA and ΔgpsB mutants repaired back to WT. divIVA and gpsB genes were inserted back to their genuine chromosomal locus in either the ΔdivIVA mutant or the ΔgpsB mutant to obtain divIVA + and gpsB + strains, respectively. (A) Cell shape of divIVA + and gpsB + cells. Phase contrast microscopy (left panel) and FM4–64 membrane staining (right panel) images of exponentially growing cells at 37°C in THY medium. Scale bar, 5 µm. (B) Growth of divIVA + and gpsB + cells compared to WT cells. Strains were grown in THY medium at 37°C in a JASCO V-630 Biospectrophotometer. The OD550 was read automatically every 10 min. (TIF) [file pgen.1004275.s001.tif]

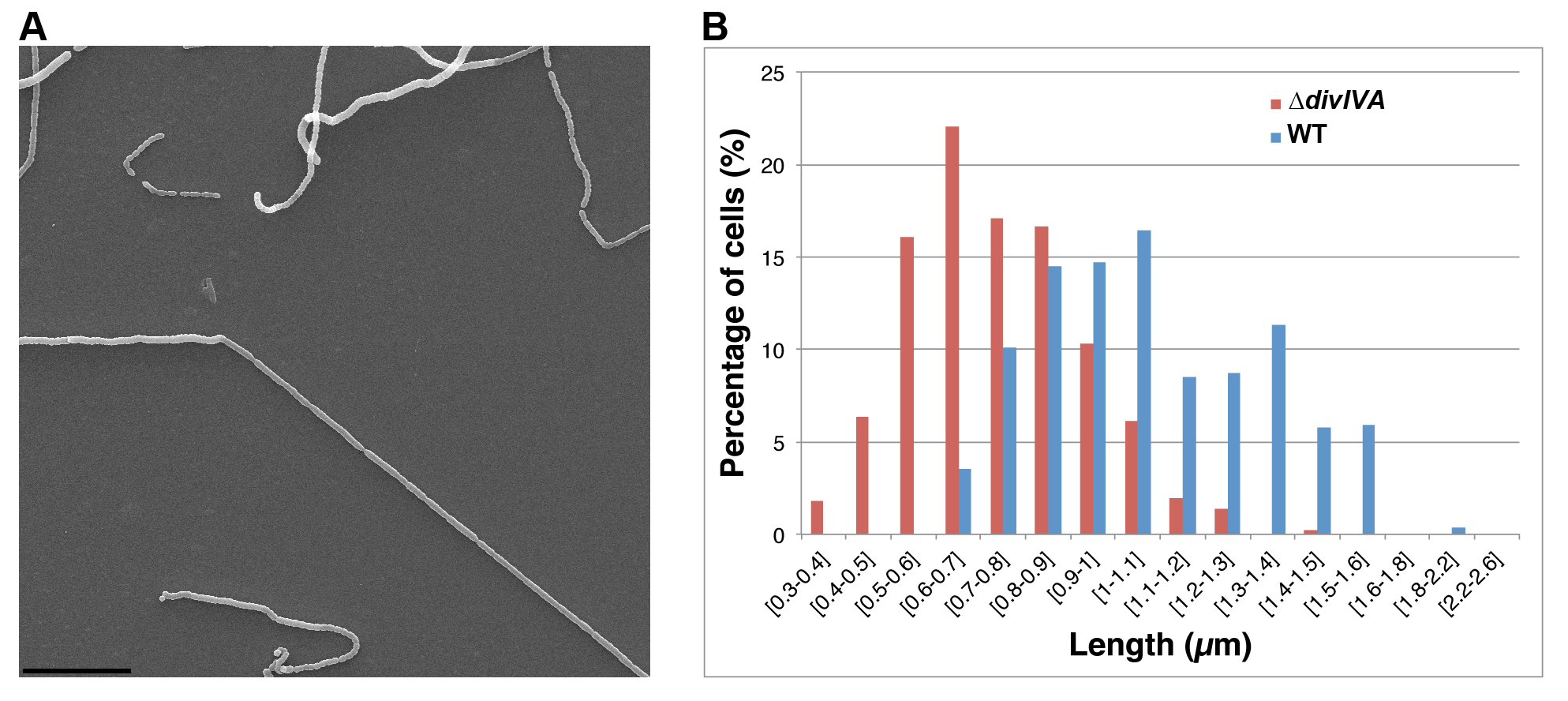

Supplement: Figure S2 — Morphology and cell length of ΔdivIVA cells. (A) ΔdivIVA cells were grown at 37°C in THY medium and observed by scanning electron microscopy. Scale bar, 10 µm. (B) Frequency of the length parameter of ΔdivIVA cells compared to WT cells. Strains were grown in THY medium at 37°C up to OD550 = 0.1. The lengths of at least 500 cells of WT and ΔdivIVA strains, based on phase-contrast images, were measured using ImageJ. (TIF) [file pgen.1004275.s002.tif]

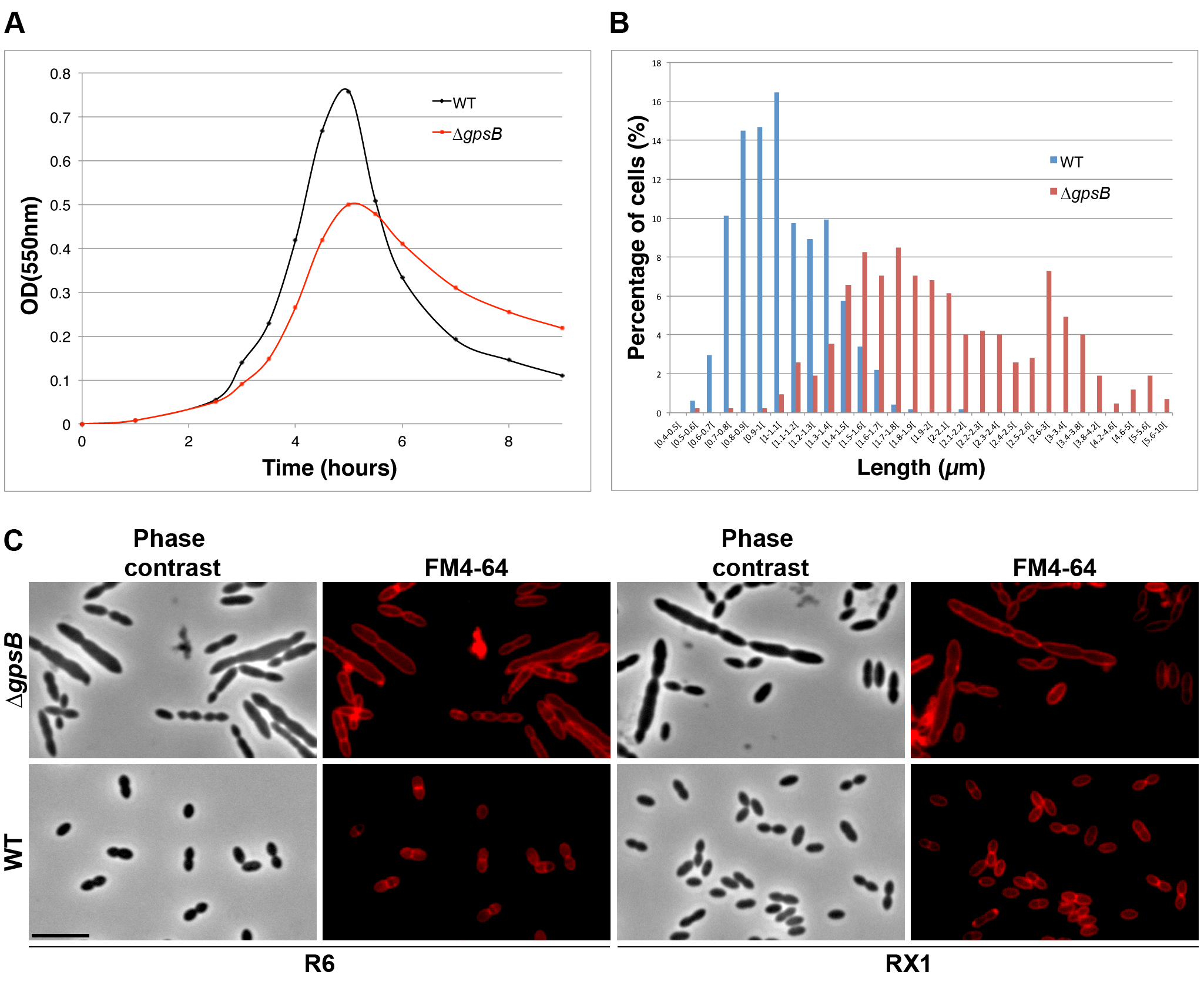

Supplement: Figure S3 — GpsB is required for S. pneumoniae growth and cell division. (A) Effect of gpsB deletion on pneumococcal growth. WT (black curve) and ΔgpsB (red curve) strains were grown in THY medium at 37°C. The OD550 was read automatically every 10 min. (B) Frequency of the length parameter of ΔgpsB cells compared to WT cells. Strains were grown in THY medium at 37°C up to OD550 = 0.1. The lengths of at least 500 cells of WT and ΔgpsB cells, based on phase-contrast images, were measured using ImageJ. (C) Phase contrast microscopy (grey) and FM4–64 membrane staining (red) of gpsB-deficient (upper row) and WT (lower row) R6 and RX1 growing cells at 37°C in THY medium. Scale bar, 5 µm. (TIF) [file pgen.1004275.s003.tif]

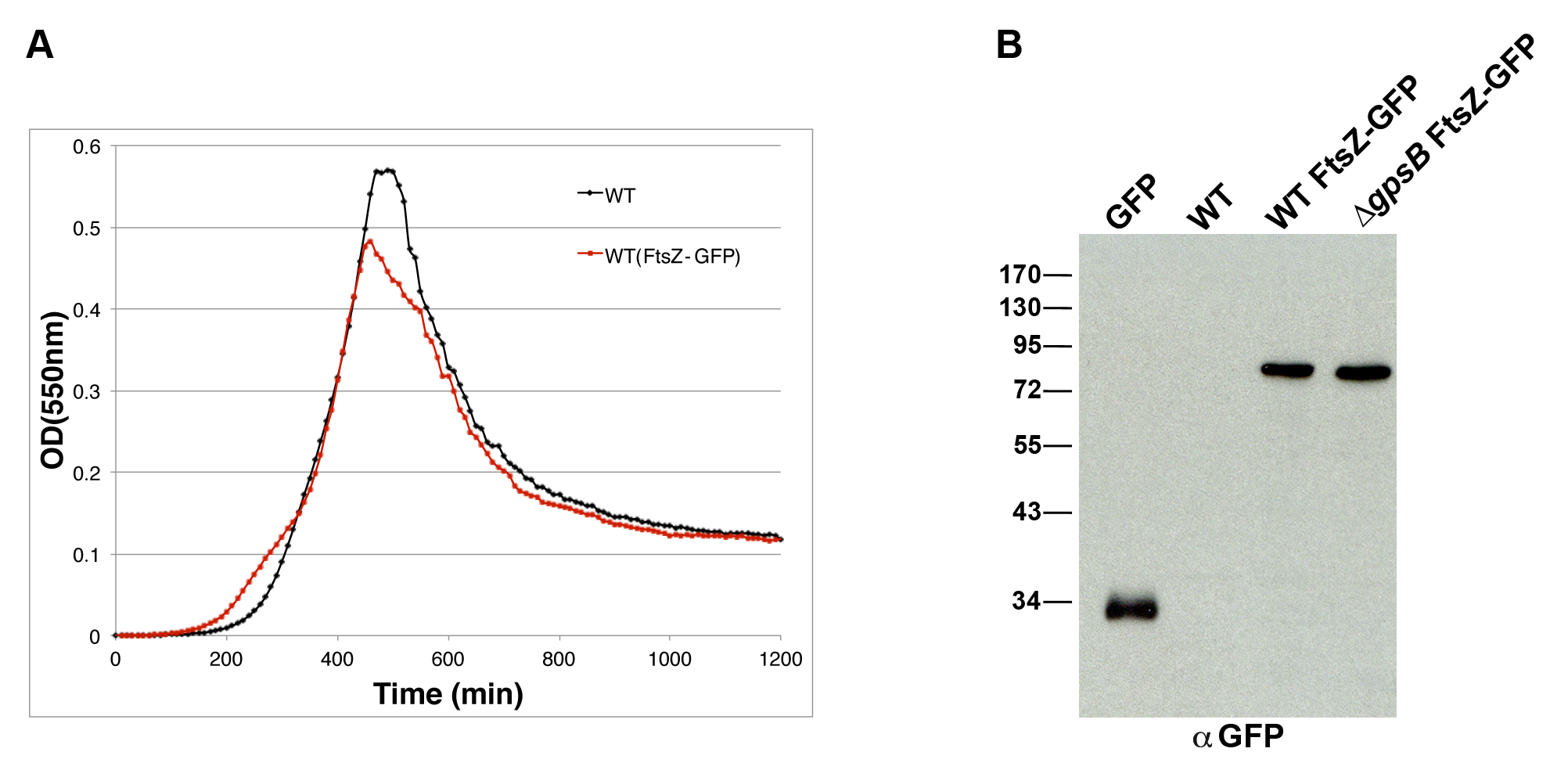

Supplement: Figure S4 — Analysis of WT cells expressing FtsZ-GFP. (A) Growth curves of WT strains expressing either FtsZ (black) or FtsZ-GFP (red) as the only source of FtsZ from its endogenous chromosomal locus grown in THY medium at 37°C. The OD550 was read automatically every 10 min. (B) Expression of the FtsZ-GFP fusion in WT and ΔgpsB cells. Cells were grown in THY medium at 37°C to OD550 = 0.3. Crude extracts (25 µg) of WT or ΔgpsB cells expressing FtsZ fused to GFP were analyzed by SDS-PAGE, electro-blotted onto a PVDF membrane and probed with anti-GFP antibodies. Purified GFP and a crude extract of WT cells not producing FtsZ-GFP were used as controls. (TIF) [file pgen.1004275.s004.tif]

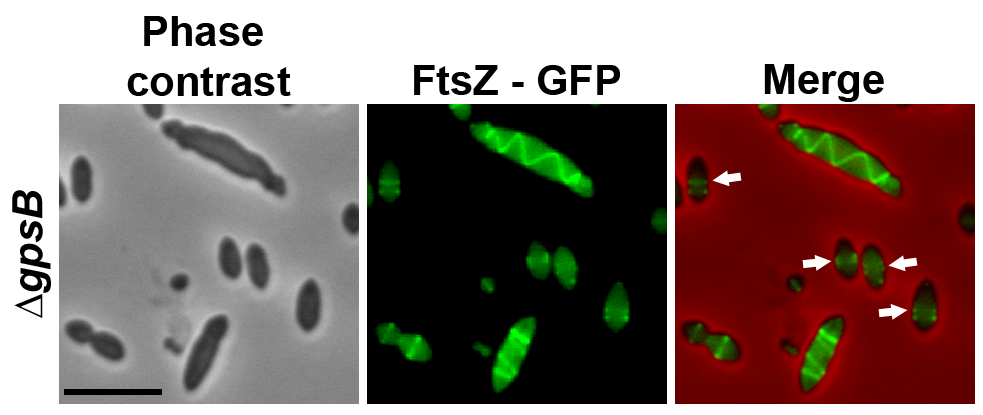

Supplement: Figure S5 — FtsZ localization in ΔgpsB cells. Same image as in Figure 3A but unprocessed. Arrows show cells without FtsZ-GFP signal in Figure 3A. Phase contrast (left), GFP fluorescent signal (middle) and overlays (right) between phase contrast (red) and GFP (green) images are shown. Scale bar, 5 µm. (TIF) [file pgen.1004275.s005.tif]

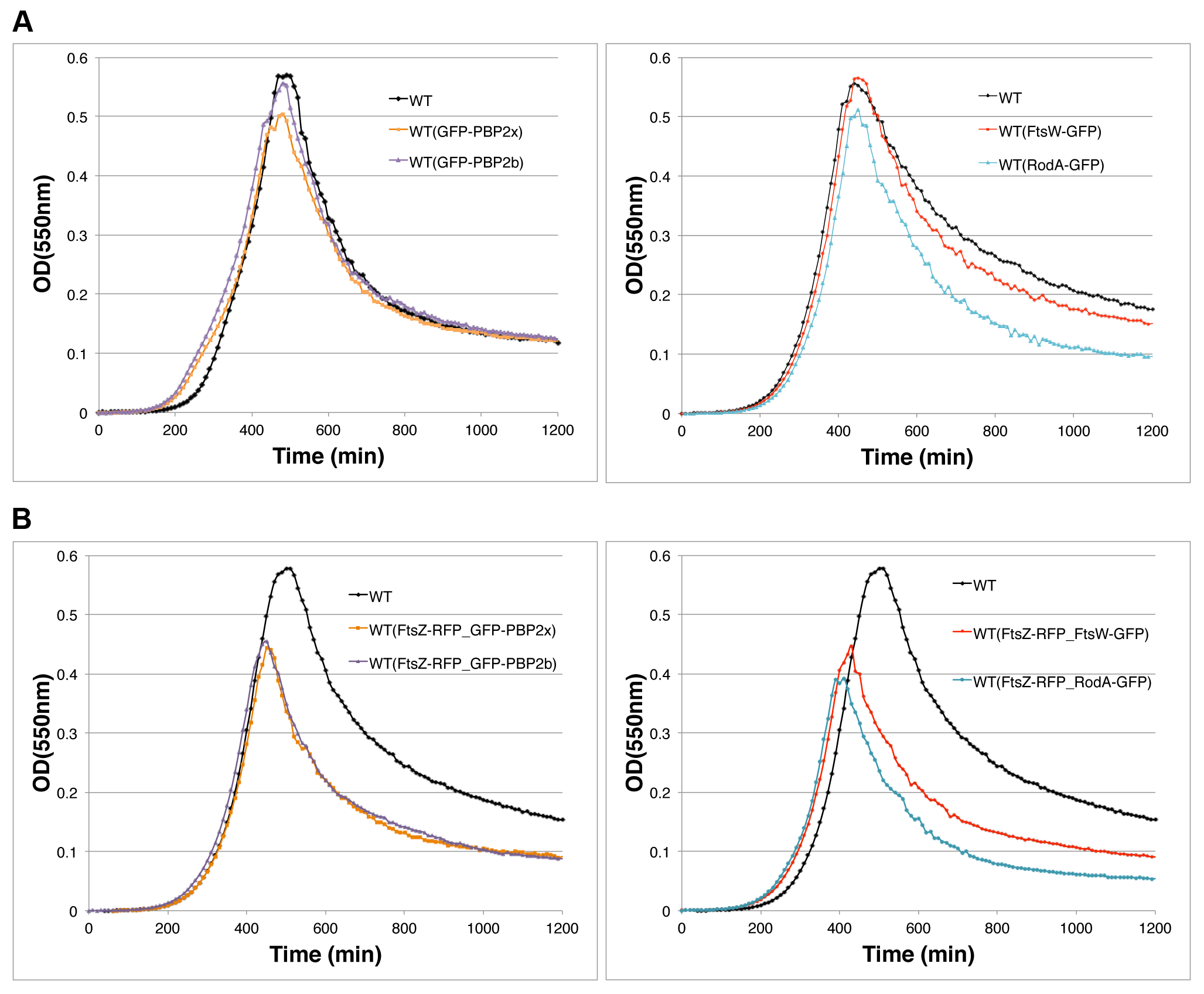

Supplement: Figure S6 — Growth curves of WT cells expressing GFP-PBP2x, GFP-PBP2b, FtsW-GFP or RodA-GFP fusions. (A) Growth curves of WT cells (black) and cells expressing either GFP-PBP2x (orange) or GFP-PBP2b (purple) (left panel), or FtsW-GFP (red) or RodA-GFP (blue) (right panel) in THY medium at 37°C. The OD550 was read automatically every 10 min. (B) Same as above but in cells also expressing the FtsZ-RFP fusion. All fusion proteins are the only source of PBP2x, PBP2b, FtsW, RodA or FtsZ in the cells. The fusion genes encoding these proteins substitute the corresponding native genes at their chromosomal locus. (TIF) [file pgen.1004275.s006.tif]

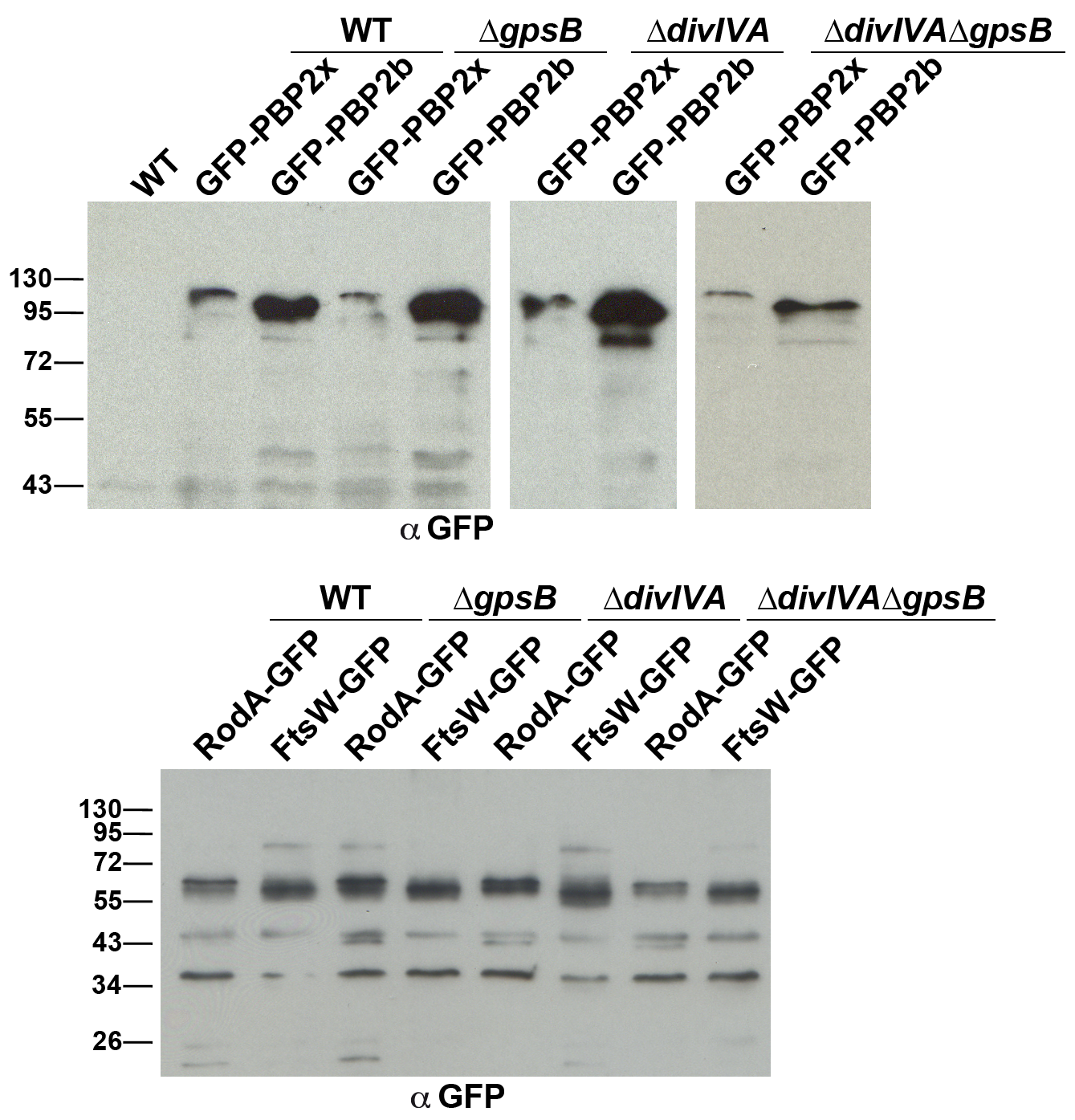

Supplement: Figure S7 — Expression of GFP-PBP2x, GFP-PBP2b, FtsW-GFP or RodA-GFP fusions. Expression of GFP-PBP2x and GFP-PBP2b fusions (upper row) and FtsW-GFP and RodA-GFP fusions (lower row) in WT, ΔgpsB, ΔdivIVA and ΔdivIVAΔgpsB strains. Cells were grown in THY medium at 37°C. Crude extracts (25 µg) were analyzed by SDS-PAGE, electro-blotted onto a PVDF membrane and probed with anti-GFP antibodies. (TIF) [file pgen.1004275.s007.tif]

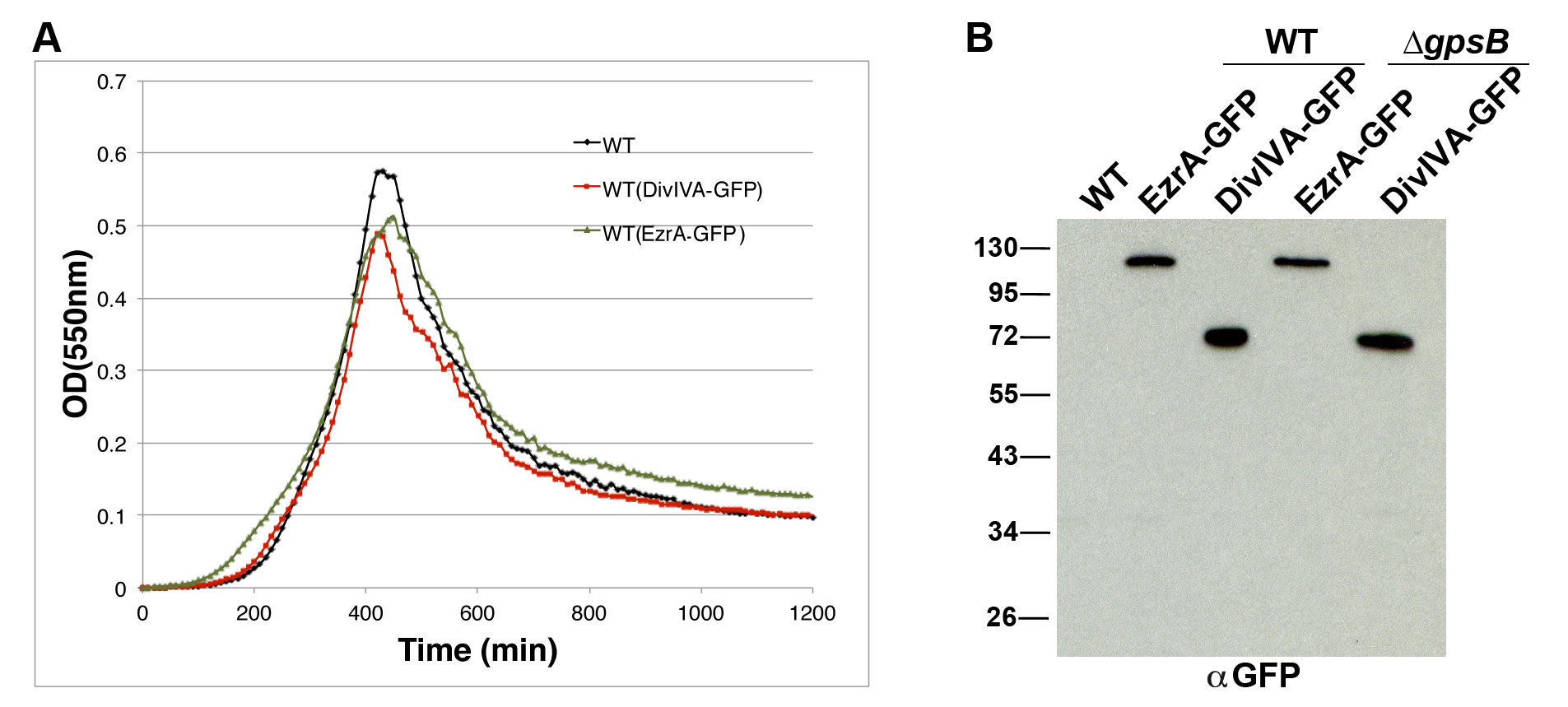

Supplement: Figure S8 — Growth curves and expression of DivIVA-GFP and EzrA-GFP fusions. (A) Growth curves of WT cells (black) and cells expressing either DivIVA-GFP (red) or EzrA-GFP (green) in THY medium at 37°C. The OD550 was read automatically every 10 min. DivIVA-GFP and EzrA-GFP were produced as the only source of DivIVA and EzrA. (B) Expression of EzrA-GFP and DivIVA-GFP fusions in WT and ΔgpsB strains. Cells were grown in THY medium at 37°C to OD550 = 0.3. Crude extracts (25 µg) of WT or ΔgpsB cells expressing either DivIVA or EzrA fused to GFP were analyzed by SDS-PAGE, electro-blotted onto a PVDF membrane and probed with anti-GFP antibodies. (TIF) [file pgen.1004275.s008.tif]

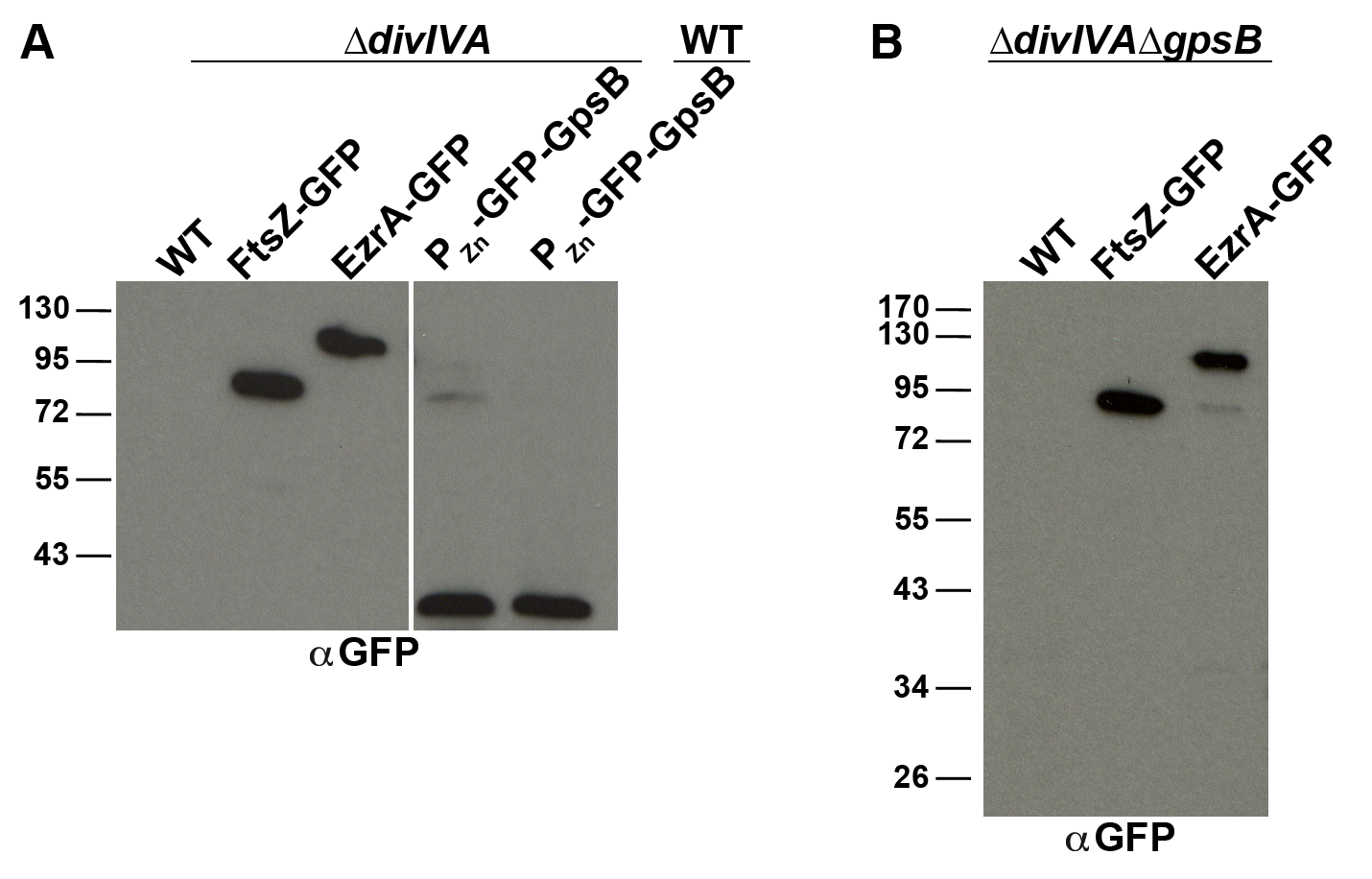

Supplement: Figure S9 — Expression of GFP fusions in ΔdivIVA and ΔdivIVAΔgpsB cells. (A) Expression of GFP-fused FtsZ and EzrA expressed as a single copy substituting the chromosomal ftsZ and ezrA genes, respectively, in ΔdivIVA strain. For GpsB, expression from the PZn promoter was assessed both in WT and ΔdivIVA strains. Crude extracts (25 µg) of WT or ΔgpsB cells expressing FtsZ fused to GFP were analyzed by SDS-PAGE, electro-blotted onto a PVDF membrane and probed with anti-GFP antibodies. A crude extract of WT untagged cells was used as control. (B) Same as above for FtsZ and EzrA GFP fusions but in ΔdivIVAΔgpsB cells. (TIF) [file pgen.1004275.s009.tif]

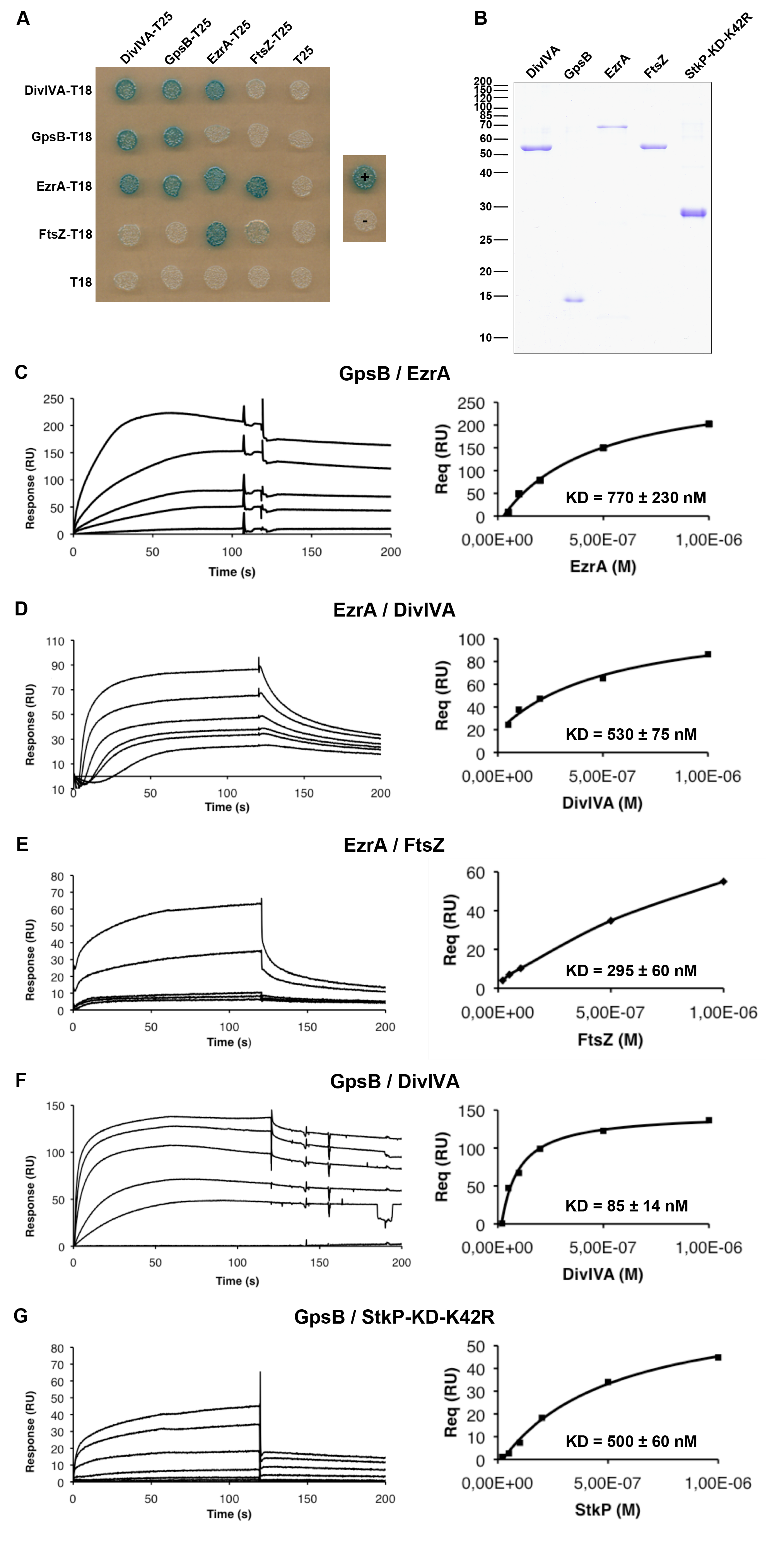

Supplement: Figure S10 — Analyses of the interactions. (A) Bacterial two-hybrid analyses. Plasmids expressing either the T18 or the T25 fragments of the adenylate cyclase protein fused to the C-terminus of DivIVA, GpsB, FtsZ and EzrA were constructed and the interactions between two candidates were assessed after co-transformation of T18- and T25-constructs in E. coli BTH101 and growth for 40 h on LB/X-Gal/IPTG plates. The blue coloration indicates positive interactions. (B) Purification of GpsB, EzrA, DivIVA, FtsZ and StkP-K42R cytoplasmic domain. Proteins were overproduced in E. coli BL21 as 6his-tagged fusion proteins. After purification using a Ni-NTA resin, purified proteins were analyzed by SDS-PAGE. (C–G) SPR analyses of interactions. (C–G, left panels) Kinetics of the interactions by Plasmon Surface Resonance (SPR) of EzrA, GpsB, DivIVA, FtsZ and StkP-K42R cytoplasmic domain. EzrA or GpsB were covalently coupled through their amino groups to the surface of a CM5 sensorchip. Increasing amounts of either GpsB (C) DivIVA (D) or FtsZ (E) were injected onto the EzrA-coupled sensorship. Similarly, increasing amounts of either DivIVA (F) or StkP (G) were injected onto the GpsB-coupled sensorship. RU: resonance units. The measurements were made in triplicate. (C–G, right panels) Non-linear regression fits to the equilibrium resonance signal (Req), obtained by extrapolation to infinite time vs. analyte concentration, used to obtain apparent equilibrium dissociation constant (KD) (see Materials and Methods). (TIF) [file pgen.1004275.s010.tif]

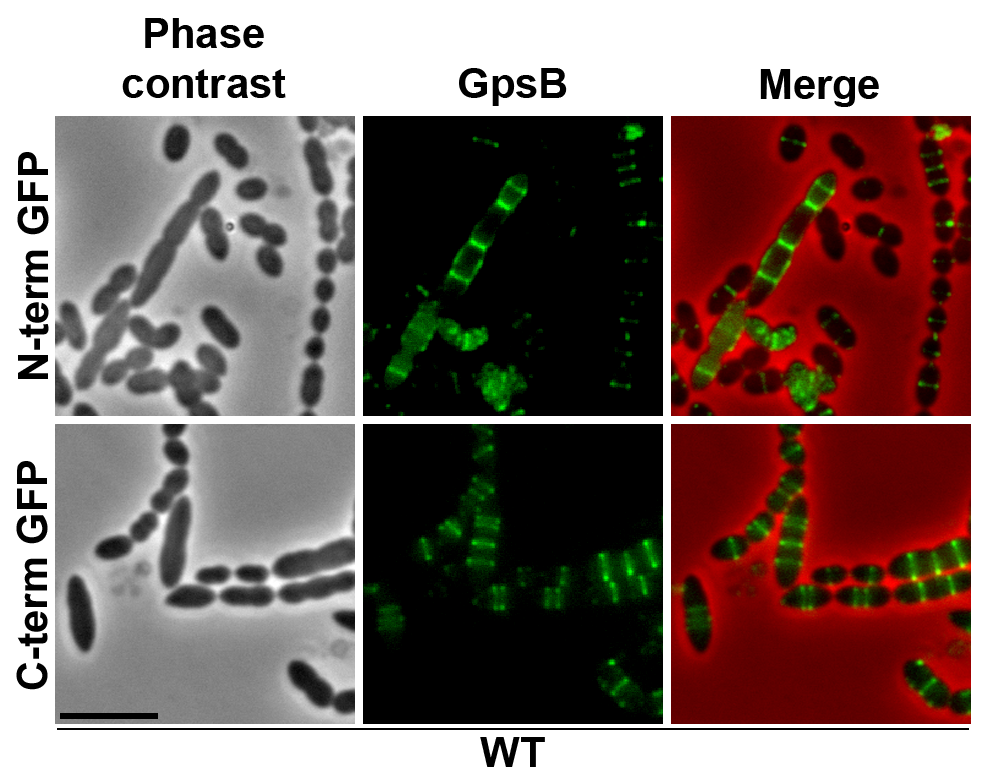

Supplement: Figure S11 — Localization of GFP fused to GpsB expressed as a single copy in WT cells. WT cells expressing either a N-terminal GFP-GpsB (upper row) or a C-terminal GpsB-GFP (lower row) fusion as a single copy substituting the chromosomal gpsB gene were grown in THY medium at 37°C. Scale bar, 5 µm. (TIF) [file pgen.1004275.s011.tif]

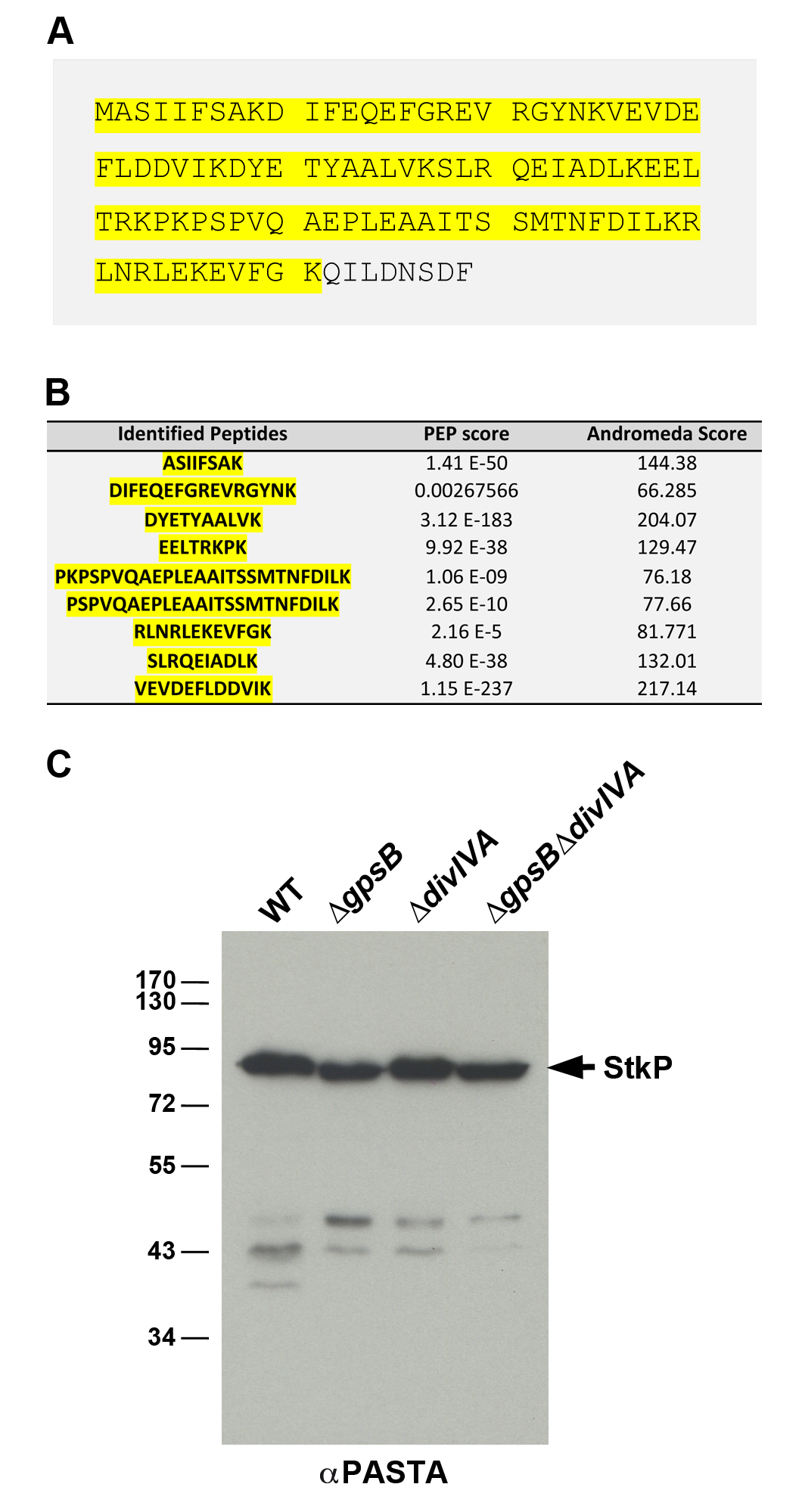

Supplement: Figure S12 — Phosphorylation of GpsB and StkP. (A) and (B) Analysis of GpsB in vivo phosphorylation in WT cells. After purification from WT cells, GpsB was analyzed by mass spectrometry (see Materials and Methods). (A) Overview into the coverage of GpsB. 93% of the amino acid sequence is identified. (B) Peptides identified are marked in yellow. MS/MS identified peptides of GpsB, along with the PEP and Andromeda scores (FDR 1%). The PEP score (Posterior probability score) represents the probability of a false hit based on the length of the peptide and the identification score that the peptide received. The smaller the PEP score, the higher the statistical probability is that the peptide was correctly identified. Searches were performed at an FDR threshold level of 1%. A search where no FDR threshold was also applied in order to ensure that no phosphorylation sites were filtered out. Peptides were identified with no phosphorylation sites detected. (C) Western immunoblot of whole-cell lysates from WT, ΔgpsB, ΔdivIVA and ΔgpsBΔdivIVA cells grown in THY at 37°C probed with anti-StkP-PASTA antibodies [14]. The same amounts (25 µg) of cell crude extracts have been loaded in all gel lanes. Arrow indicates the expression signal detected for StkP. (TIF) [file pgen.1004275.s012.tif]
