# Supplementary material for: Interplay of the Serine/Threonine-Kinase StkP and the Paralogs DivIVA and GpsB in Pneumococcal Cell Elongation and Division
Source: PLoS Genet. 2014 Apr 10;10(4):e1004275. doi: 10.1371/journal.pgen.1004275 (PMC3983041; doi:10.1371/journal.pgen.1004275)
Supplement: Text S1 — Supplemental Materials and Methods. Exhaustive description of the procedure used for allelic replacement mutagenesis and construction of mutant strains. (PDF) [file pgen.1004275.s017.pdf]

Text S1:

Supplemental Materials and Methods

Exhaustive description of the procedure used for allelic replacement mutagenesis and construction of mutant strains.

All strains and primers are indicated in Table S3 and S4, section 1, respectively. The nucleotide sequences of all final PCR DNA fragments were checked to ensure error-free amplification.

To construct pneumococcus mutants (gene deletions, *gfp*- or *rfp*-fusions), we used a two-step procedure, based on a bicistronic *kan-rpsL* cassette called Janus. This procedure allows the replacement of a gene by a cassette and subsequent deletion or substitution of the cassette by a mutated allelic form at the gene chromosomal locus. Briefly, the Janus cassette is either used to replace the gene of interest or inserted at either its 5' or 3'-end. Both options confer resistance to kanamycin and dominant streptomycin sensitivity in the wild-type streptomycin-resistant R800 *rpsL1* strain (Kan^R-Str^S). Then, any DNA fragments flanked on each end by sequences homologous to the upstream and downstream regions of the gene of interest are used to transform Kan^R-Str^S strains in order to obtain the expected nonpolar markerless mutant strains. Chromosomal DNA of the R800 strain was used as template unless stated otherwise.

To construct strains expressing C-terminus GFP fusions (DivIVA, EzrA, FtsZ, GpsB and RodA), the Janus cassette was first inserted at the 3' end of the genes at their chromosomal locus. To obtain the Janus cassette, we used the primer pair A/B and chromosomal DNA of the R1226 strain as template. Then, the upstream+gene and the downstream regions of the gene of interest were PCR amplified using primer pairs 1/2 and 3/4, respectively. The DNA fragment 1-2 and the Janus cassette were used as template in a fusion PCR using the primer

pair 1/B. The obtained DNA fragment was used together with the DNA fragment 3-4 for another fusion PCR using the primer pair 1/4. The resulting PCR product was used to transform WT and mutant strains. Then, we amplified the *gfp* gene with primer pair C/D using pUC57-*gfp* as template (45). Another round of PCR using primer pairs 1/5 and 6/4 allowed the amplification of the upstream+gene and the downstream regions of the gene of interest. A PCR fusion was then performed using primer pair 1/D and both *gfp* and the DNA fragment 1-5 as templates. The obtained DNA product was used as template together with the DNA fragment 6-4 and the primer pair 1/4 for another round of PCR fusion. The *ftsW-gfp* DNA fragment was obtained using the primer pair 1/4 and chromosomal DNA of the R3677 strain as template. The same procedure was applied to obtain *ftsZ* fused to *rfp* using primers 6-R, G, and H, instead of 6, C, and D, respectively (the *rfp* gene was obtained by PCR amplification with primer pair G/H and pHK096 as template (15).

The resulting PCR products were used to transform Kan^R-Str^S strains previously obtained and containing the Janus cassette fused to the gene of interest. The same procedure was applied to construct strains expressing N-terminus GFP fusions (for PBP2b, and GpsB), using primers 2', 3', 5', 6', E, and F, instead of primers 2, 3, 5, 6, C, and D, respectively. The *gfp-pbp2x* DNA fragment was obtained using the primer pair 1/4 and R3676 strain as template. Markerless transformants were re-streaked to single colonies and correct integration at the chromosomal locus was verified by PCR. They express GFP fused either at the N-terminus or at the C-terminus of proteins of interest.

The same strategy was used to delete *pbp2x, pbp2b, rodA* and *ftsW*. The upstream and the downstream gene regions were amplified using primer pairs 1/7 and 8/4. The two DNA fragments were then fused by PCR using primer pair 1/4, and the resulting DNA fragment 1-4 was used to transform Kan^R-Str^S strains previously obtained in which *pbp2x, pbp2b, rodA* or *ftsW* are fused to Janus. This last step was unsuccessful.

We also constructed a mero-diploid strain carrying the *gfp-gpsB* fusion under control of the zinc-inducible P_{Zn} promoter at the non-essential *bgaA* locus. For this, we transformed the WT strain with the P_{Zn}-*gfp-gpsB* plasmid (see construction of plasmids).

To delete *divIVA* and/or *gpsB*, these genes were first replaced by the Janus cassette at their chromosomal locus. For this, the upstream and downstream gene regions of *divIVA* and/or *gpsB* were PCR amplified using primer pairs 1/2' and 3/4, respectively. The Janus cassette was then fused by PCR to the DNA fragment 1-2' using the primer pair 1/B. Another round of PCR was performed to fuse the DNA fragment 1-B to the DNA fragment 3-4 using the primer pair 1/4. The resulting DNA product was used to transform the strain of interest. In these strains, *divIVA* and *gpsB* are replaced by the Janus cassette. To obtain markerless mutants, the upstream and downstream regions of either *divIVA* or *gpsB* genes were amplified again but with primer pairs 1/7 and 8/4, respectively. The two fragments were PCR fused using the primer pair 1/4 and the resulting DNA product was used to transform the Kan^R-Str^S strains previously obtained in which *divIVA* or *gpsB* were replaced by the Janus cassette. To exclude the presence of suppressive mutations distorting our observations, we also repaired Δ*divIVA* and Δ*gpsB* strains back to the WT strain. For that, Kan^R-Str^S strains in which either *divIVA* or *gpsB* were replaced by the Janus cassette were transformed by a wild type DNA product containing *divIVA* or *gpsB*, respectively. These DNA products were obtained by PCR amplification using the corresponding primer pairs 1/4 (Table S4) and WT chromosomal DNA strain as template. This last step has not been done in *gpsB*::*kan-rpsL* in R6 and RX1 strains because of the absence of the *rpsL1* allele in these strains.

The strain *gpsB*::*kan-rpsL* was also used to obtain the strain producing GpsB fused to a 6-Histidine tag at its C-terminal end at the chromosome locus. For that, the upstream+ *gpsB* region and the downstream region of *gpsB* were PCR amplified using primer pairs 1/6His(-) and 6His(+)/4, respectively. Then, the two resulting PCR DNA fragments were fused by PCR

using primer pair 1/4. The resulting DNA product was used to transform the Kan^R-Str^S strain previously obtained in which *gpsB* was replaced by the Janus cassette.

The *gfp-stkP* and *phpP-stkP*::*kan-rpsL* DNA fragments were obtained by PCR using primer pair 1/4 and chromosomal DNA of the WT-*gfp-stkP* strain and the Spn8 strain as template. The obtained PCR products were used to transform the markerless Δ*gpsB,* Δ*divIVA* and Δ*divIVA*Δ*gpsB* strains.
